# PRE-1 Revealed Previous Unknown Introgression Events in Eurasian Boars during the Middle Pleistocene

**DOI:** 10.1093/gbe/evaa142

**Published:** 2020-07-10

**Authors:** Pengju Zhao, Heng Du, Lin Jiang, Xianrui Zheng, Wen Feng, Chenguang Diao, Lei Zhou, George E Liu, Hao Zhang, Yangzom Chamba, Qin Zhang, Bugao Li, Jian-Feng Liu

**Affiliations:** National Engineering Laboratory for Animal Breeding; Key Laboratory of Animal Genetics, Breeding and Reproduction, Ministry of Agriculture; College of Animal Science and Technology, China Agricultural University, Beijing, China; National Engineering Laboratory for Animal Breeding; Key Laboratory of Animal Genetics, Breeding and Reproduction, Ministry of Agriculture; College of Animal Science and Technology, China Agricultural University, Beijing, China; Institute of Animal Science, Chinese Academy of Agricultural Sciences (CAAS), Beijing, China; National Engineering Laboratory for Animal Breeding; Key Laboratory of Animal Genetics, Breeding and Reproduction, Ministry of Agriculture; College of Animal Science and Technology, China Agricultural University, Beijing, China; National Engineering Laboratory for Animal Breeding; Key Laboratory of Animal Genetics, Breeding and Reproduction, Ministry of Agriculture; College of Animal Science and Technology, China Agricultural University, Beijing, China; National Engineering Laboratory for Animal Breeding; Key Laboratory of Animal Genetics, Breeding and Reproduction, Ministry of Agriculture; College of Animal Science and Technology, China Agricultural University, Beijing, China; National Engineering Laboratory for Animal Breeding; Key Laboratory of Animal Genetics, Breeding and Reproduction, Ministry of Agriculture; College of Animal Science and Technology, China Agricultural University, Beijing, China; Animal Genomics and Improvement Laboratory, BARC, USDA-ARS, Maryland; National Engineering Laboratory for Animal Breeding; Key Laboratory of Animal Genetics, Breeding and Reproduction, Ministry of Agriculture; College of Animal Science and Technology, China Agricultural University, Beijing, China; College of Animal Science and Technology, Tibet Agriculture and Animal Husbandry College, Linzhi, Tibet, China; National Engineering Laboratory for Animal Breeding; Key Laboratory of Animal Genetics, Breeding and Reproduction, Ministry of Agriculture; College of Animal Science and Technology, China Agricultural University, Beijing, China; College of Animal Science and Technology, Shandong Agricultural University, Taian, Shandong, PR China; Department of Animal Sciences and Veterinary Medicine, Shanxi Agricultural University, Taigu, China; National Engineering Laboratory for Animal Breeding; Key Laboratory of Animal Genetics, Breeding and Reproduction, Ministry of Agriculture; College of Animal Science and Technology, China Agricultural University, Beijing, China

**Keywords:** pigs, transposable elements, PRE1-SS, population admixture and divergence

## Abstract

Introgression events and population admixture occurred among *Sus* species across the Eurasian mainland in the Middle Pleistocene, which reflects the local adaption of different populations and contributes to evolutionary novelty. Previous findings on these population introgressions were largely based on extensive genome-wide single-nucleotide polymorphism information, ignoring structural variants (SVs) as an important alternative resource of genetic variations. Here, we profiled the genome-wide SVs and explored the formation of pattern-related SVs, indicating that PRE1-SS is a recently active subfamily that was strongly associated with introgression events in multiple Asian and European pig populations. As reflected by the three different combination haplotypes from two specific patterns and known phylogenetic relationships in Eurasian boars, we identified the Asian Northern wild pigs as having experienced introgression from European wild boars around 0.5–0.2 Ma and having received latitude-related selection. During further exploration of the influence of pattern-related SVs on gene functions, we found substantial sequence changes in 199 intron regions of 54 genes and 3 exon regions of 3 genes (*HDX*, *TRO*, and *SMIM1*), implying that the pattern-related SVs were highly related to positive selection and adaption of pigs. Our findings revealed novel introgression events in Eurasian wild boars, providing a timeline of population admixture and divergence across the Eurasian mainland in the Middle Pleistocene.

**Significance**

PRE-1 as active subfamilies of transposable elements (TEs) can spreading out copies of themselves in the genome and have great impact on the genome structure. Here, we detected a particular 42-Mb TE hotspot region formed by PRE-1 on chromosome X of *Sus scrofa* with distinct distribution patterns across Eurasian boar populations. Three genes (*HDX*, *TRO*, and *SMIM1*) in this region were found with exon structure differences triggered by PRE-1 between Eurasian boar populations. More intriguingly, PRE-1 can create a clear route map of population admixture occurred in Asian and European boars during the Middle Pleistocene epoch. Our findings provided novel evidence on introgressions among wild boar populations and put new insights into the role of TEs in porcine genome evolution.

## Introduction

*Sus scrofa* originated from the genus *Sus* ∼3–4 Ma and initially appeared in Southeast Asia (Groenen 2016). After the divergence of the Sumatra wild boar from the Eurasian wild boar ∼1.5–2 Ma (Frantz et al. 2013), two independent wild boar populations diverged from Southeast Asia and invaded the entire Eurasian mainland around 1 Ma (Groenen et al. 2012). Since then, multiple admixture and divergence events occurred among *Sus* species inhabiting Eurasian mainland, either by natural dispersal or by human intervention. For example, within Asia, obvious divergence between Northern and Southern Chinese *S. scrofa* populations occurred around 0.6 Ma (Frantz et al. 2013). Tibetan pigs were likely admixed with neighboring lowland breeds for developing a complex biological adaptation mechanism (Ai et al. 2014). In Europe, wild boars experienced more bottlenecks because of the last glacial period that resulted in a notably lower genetic diversity compared with the Asian wild boar (Frantz et al. 2013). It is notable that most of these findings were inferred merely based on genomic single-nucleotide polymorphism (SNP) markers, while the structural variants (SVs), such as transposable elements (TEs) that are likely to contribute to adaptive evolution have been largely ignored (Sotero-Caio et al. 2017).

The advent of genome-scanning technologies provided a deep understanding of SVs in the human genome (Feuk et al. 2006; Stewart et al. 2011; Sudmant, Rausch, et al. 2015; Chiang et al. 2017). The utility of SVs as an ancestry informative marker facilitated the recognition patterns of human evolution, admixture, and migration (Cordaux and Batzer 2009; Lupski 2015; Sudmant, Mallick, et al. 2015). Regarding pig genomes, we previously identified an obvious SV homozygosity hotspot region of 35 Mb with extremely low recombination in the X chromosome, where two specific SV patterns totally segregated in European and Asian pig breeds (Zhao et al. 2016). It was reported that this large X-linked fragment is potentially associated with the latitude-related natural selection and interspecies introgression (Ai et al. 2015); however, there is still a lack of explanation for how and when this pattern formed.

TEs, the potential cause of SV by spreading out copies of themselves in the genome, are being gradually recognized as valuable evolutionary markers in populations and species (Fedoroff 2012; Bourque et al. 2018). Compared with SNPs, TEs are highly stable polymorphisms and are usually shared between individuals with identity-by-descent (Rishishwar et al. 2015). So far, various specific TEs have been recognized in several organisms along with their corresponding function, especially in the evolution of primates and rodents (Liu et al. 2009; Doronina et al. 2019). Studies on the human genome showed that TEs not only play an important role in the regulation of protein-coding and noncoding parts of the genome but also have evolutionary potential in their introns as a way of forming new exons (Ule 2013). In the swine genome, sequence homology analyses indicated that one type of SINE, the PRE-1 element, shared a common ancestor (7SL RNA) with primate SINEs. This suggests that porcine TEs likely have a function similar to that of human TEs, such as biochemical properties and mobilization mechanisms (Deininger 2011; Kramerov and Vassetzky 2011).

We speculated that the previously detected 35 Mb X chromosome-related SV homozygosity region was likely caused by TE. Combining the results of a previous study (Ai et al. 2015), we also infer that this region was related to the population adaption and introgression between Asian and European pigs. Based on this hypothesis, we herein propose a novel assembly-based strategy to precisely identify whole-genome SVs (supplementary fig. S1, Supplementary Material online). We obtained a total of 13,310,147 SVs in 13 genome assemblies (including European pig breeds, Asian pig breeds, and a crossbreed pig). Among these identified SVs, we determined a total of 47,808 SVs (0.36%) were haplotype specific while enriched in the X chromosome. Specifically, 34,171 SVs segregate between Asian and European originated pigs and 13,637 SVs were segregated between Asian-South pigs and non-Asian-South pigs. Furthermore, 73.7% out of these haplotype-specific SVs (*n* = 35,234) in the X chromosome were in the 42-Mb region which contained the 35-Mb region defined in our previous study (Zhao et al. 2016). These enriched SVs (length >100 bp) were further validated by large-scale next generation sequencing (NGS) data of 316 individuals (supplementary fig. S1, Supplementary Material online). Intriguingly, among these SVs harbored in the 42-Mb region, 63.7% belonged to LINE and SINE families and 40.4% of which were fell into the category of PRE-1 subfamily. Screening these pattern-related TEs based on known evolutionary relationships of *S. scrofa*, we inferred the origins and the latest activity periods of pattern-related TEs in the special landscape of chromosome X. We propose a novel hypothesis for two distinct patterns in the chromosome X carried by different pig breeds, which clearly explains a succession of previously unknown introgression and speciation events in Eurasian boars during the Middle Pleistocene.

## Materials and Methods

### Genome-Wide Detection of SVs from Assembly Genome

To perform high-confidence SV detection (indels, insertions, and deletions) within the pig genome, we chose the assembly-based method to replace the common resequencing approach in which second-generation sequencing reads are aligned to a single reference genome. The assembly-based method mainly depends on the identification of aligned breakpoints from the full alignment of de novo sequenced genomes against a reference genome (Nattestad and Schatz 2016). Therefore, a total of 14 genome assemblies (supplementary table S1, Supplementary Material online) from 13 pig breeds were downloaded from the NCBI database (ftp://ftp.ncbi.nlm.nih.gov/genomes) to compare SV polymorphisms between pig breeds.

In our assembly-based SV detection pipeline, we first performed a quality assessment of all the genome assemblies (supplementary table S1, Supplementary Material online) and selected the highest-quality genome as the reference genome: the Duroc genome (*S. scrofa* 11.1), a European breed, which had the largest minimum contig length needed to cover 50% of the genome (N50). The other 13 pig genomes were used as the assembled genome to detect deletions and insertions.

Then, we identified the highly conserved regions between the Duroc genome and each assembled genome using the nucmer program of MUMmer 4 (http://mummer.sourceforge.net/; last accessed July 15, 2020) (Delcher et al. 2002). The parameters for the nucmer program were −*l* = 100 (the minimum length of a maximal match) and −*c* = 500 (the minimum length of a cluster, a cluster consists of two or more adjacent maximal matches). Next, the alignments from the nucmer program were used to identify high-confidence SVs in each contig of assembled genome relative to the Duroc genome by Assemblytics tool (http://assemblytics.com/; last accessed July 15, 2020). Finally, to facilitate comparison of genetic variations among the various genomes, all SVs from 13 pig genomes were merged as a nonoverlapping SV data set (unique genomic locations on Duroc genome).

### Classification of SVs Based on Two Specific Patterns

**Fig. 1:**
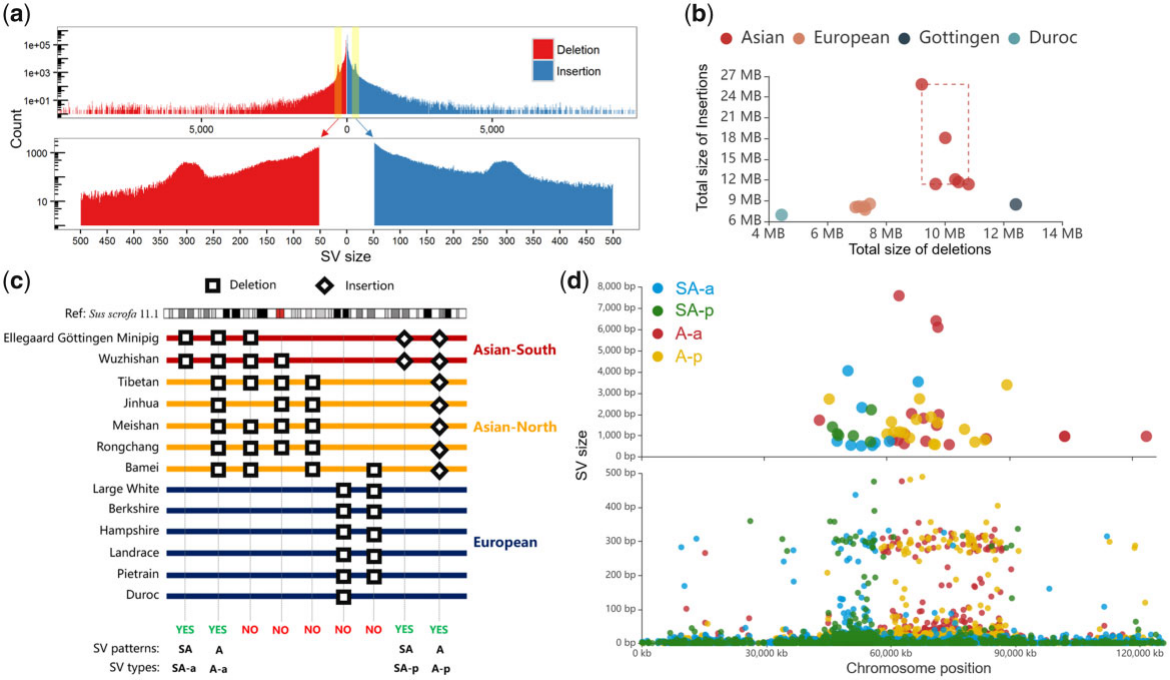
Genome-wide identification of SVs between Chinese and European pigs. (*a*). Genomic distribution of high-precision SVs in 13 pig genome assemblies. The bar plot indicates the frequency distribution of SV size for all insertions and deletions ranging between 1 bp and 10 kb. The *x* axis represents the SV size and the *y* axis indicates the count of SV. (*b*) Genomic differences between 13 pig genome assemblies and the Duroc reference genome are reflected in the coordinate axis, whereas the *x* axis and *y* axis represent the total size of deletions and insertions, respectively. (*c*) Classification strategy of pattern-related SVs. Four types of SVs were extracted from the nonoverlapping SV data set. The “yes” and “no” represent whether the SVs meet the pattern criteria and were retained. (*d*). Scatter diagram displaying the distribution of pattern-related SVs on the X chromosome. The *x* axis represents the chromosome position and the *y* axis represents the size of the SVs. The upper scatter plot indicates an SV size of >500 bp, whereas the lower chart indicates an SV size of <500 bp.

As shown in previous studies (Ai et al. 2015; Zhao et al. 2016), there were two specific patterns on chromosome X including pattern A and SA. Pattern SA represented about 14-Mb region (position: 44.0–58 Mb) that showed genomic structural difference between South Asia and non-South Asia pig breeds, and pattern A represented about 34-Mb region (position: 58–92 Mb) that showed genomic structural difference between European and Asian pig breeds. To confirm these two specific patterns in the view of SV, we extracted four types of SVs (belong to two specific patterns) from the nonoverlapping SV data set using in house Perl script. As shown in figure 1*c*, the detailed classification described as follows:

*SVs from pattern A*. The different genomic sequence between European and Asian pig breeds, including A-a type of SV (Asian-absence, represents the genomic sequences only found in all European breeds and not in Asian pig breeds) and A-p type of SV (Asian-presence, represents the genomic sequences only found in all Asian pig breeds and not in European breeds).

*SVs from pattern SA*. The different genomic sequence between South Asia (Wuzhishan pig and Ellegaard Gottingen minipig) and non-South Asia pig breeds, including SA-a type of SV (South Asia-absence, represents the genomic sequences only found in all non-South Asia pig breeds and not in South Asia pig breeds) and SA-p type of SV (South Asia-presence, represents the genomic sequences only found in all South Asia pig breeds and not in non-South Asia pig breeds).

### Validation of X Chromosome-Related Specific SVs Using Large-Scale NGS Data

We performed the large-scale NGS-based validation to verify the same patterns for these specific SVs in other pig populations or breeds. A total of 316 individuals from 65 different pig breeds were used in this study, of which 57 individuals were uploaded to NCBI under BioProject ID PRJNA378496. The remaining 259 individuals were downloaded from the NCBI database. The accession numbers for all pigs and their classification are provided in supplementary table S2, Supplementary Material online.

Then, quality control was conducted for the each raw NGS data using the NGSQC Toolkit (Patel and Jain 2012): 1) reads with adapter sequence were deleted, 2) reads with more than 30% low quality bases (quality value ≤20, or N bases) were discarded, and 3) for each read, the low quality 3′ end with base quality scores lower than 20 was trimmed.

**Fig. 2:**
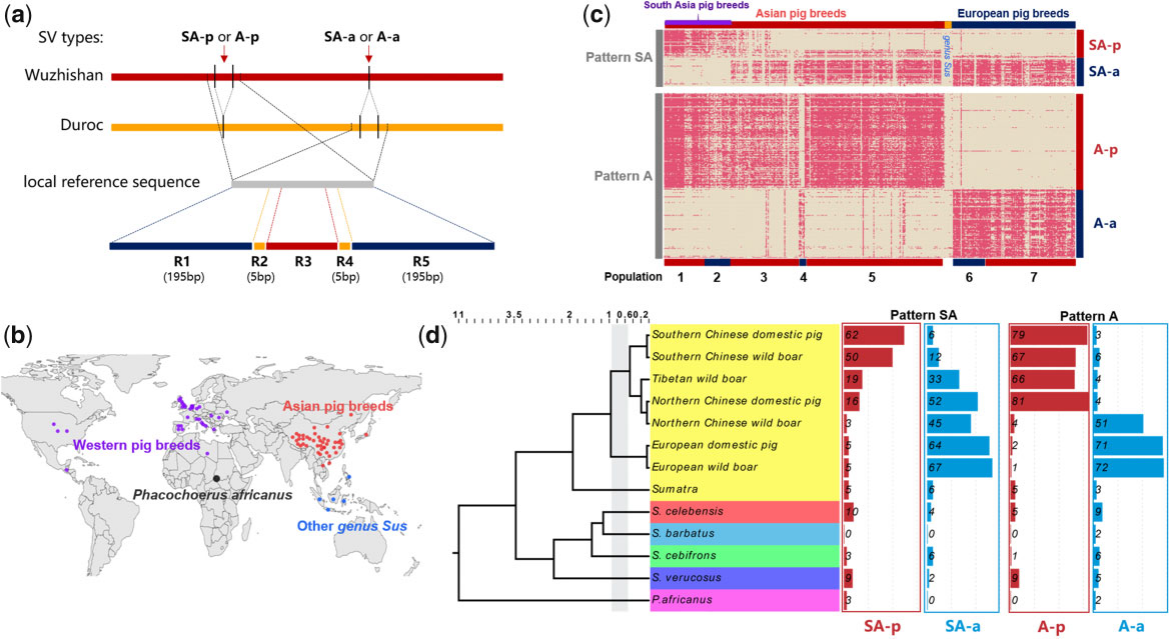
Verification of 327 specific pattern-related SVs using large-scale NGS data. (*a*) The local assembly methods to validate the pattern-related SVs for each pig. Five regions were defined for each SVs: R1 (left 195 bp from R2 region), R2 (left 5 bp from SV breakpoint), R3 (SV boundaries), R4 (right 5 bp from SV breakpoint), and R5 (right 195 bp from R4 region). (*b*) The 316 individuals from 65 different pig breeds distributed throughout Eurasia. (*c*) A heatmap for pattern-related SVs among 316 individuals from four major areas. The red highlight represents the SV region of a specific individual that was successfully covered by its sequencing reads. For example, A-a type with pattern A which can be covered by the sequencing reads of a European breed is marked in red; however, the sequence of A-a type with pattern A which cannot be covered by the sequencing reads of an Asian breed is not marked. Populations 1–7 represent Southern Chinese wild boar, Southern Chinese domestic pig, Tibetan wild boar, Northern Chinese wild boar, Northern Chinese domestic pig, European domestic pig, and European wild boar, respectively. (*d*) Phylogenetic tree of different species within the genus *Sus*, where bar plots indicate the percentage of the pattern-related SVs within different pig species/breeds.

Next, for each specific SV, we extracted local references from the reference genome based on their genomic location (SV boundaries ±200 bp). As shown in figure 2*a*, the A-a and SA-a types of SV, Duroc (*S. scrofa* 11.1) genome as the reference genome was used to represent the Asian-absence or South Asia-absence sequences. However, the A-p and SA-p types of SV, the Wuzhishan (Scaffold N50 = 5.8 Mb, highest-quality pig genome in South Asia) genome was used as the reference to represent the Asian-presence or South Asia-presence sequences. Then, the corrected NGS reads were mapped to each local reference using mrsfast (https://github.com/sfu-compbio/mrsfast; last accessed July 15, 2020) software version 3.4.0 with default parameters. The resulting alignments were further processed with our customized criteria to determine whether the SV existed: 1) Definition of R1–R5 regions (fig. 2*a*): R1 (left 195 bp from R2 region), R2 (left 5 bp from SV breakpoint), R3 (SV boundaries), R4 (right 5 bp from SV breakpoint), and R5 (right 195 bp from R4 region). 2) At least 60% of the R3 region was covered by the corrected NGS reads, 3) both of R2 and R4 regions were completely covered by the corrected NGS reads, and 4) at least 50% of R1 and R5 regions were covered by the corrected NGS reads.

### TEs in the Pig Genome and Their Relationship with Specific SVs

The whole TE boundaries and family relationships of the pig genome were identified by aligning the Duroc (*S. scrofa* 11.1) genome against the pig-specific library from Repbase update (https://www.girinst.org/repbase/; last accessed July 15, 2020) using RepeatMasker version 4.0.6 (http://www.repeatmasker.org; last accessed July 15, 2020) with an RMBlast engine.

The sequences of the R3 region (fig. 2*a*) from 327 specific SVs (length >100 bp) were extracted and clustered by multiple sequence alignment reverse complementation using MAFFT software (Katoh and Standley 2013) with default parameters. The clustered sequences of the R3 region were then aligned against the TE of pig genome using BlastN version 2.2.31+ with default parameters, and the repeat family to which these clustered sequences belonged to was defined by their best high-qualified match between TEs.

### Phylogenetic Relationships of Specific SV-Related TEs

PRE (SINE) and L1 (LINE) families of TE from pig-specific library (Repbase update) were selected to reflect the phylogenetic relationships of specific SV-related TEs. A total of 15 PRE and 8 L1 families with 275 and 7,567 loci in the final data set were used to construct phylogenetic trees and haplotype networks. Phylogenetic tree analysis was conducted in MEGA7 (https://www.megasoftware.net/; last accessed July 15, 2020) by applying Neighbor-Joining and BIONJ algorithms to a matrix of pairwise distances estimated using the maximum composite likelihood approach. Haplotype network construction was performed to visualize the genotype relationship between TEs from same families using PopART version 1.7 (http://popart.otago.ac.nz/index.shtml; last accessed July 15, 2020).

### Classification of SV-Related TE Subfamilies and Prediction of Their Activity Periods

To predict the activity time of SV-related TE, we carried out the TE subfamily classification and their divergence time estimation. In the TE subfamily classification, sequence clustering was first performed for all 15 PRE and 8 L1 consensus sequences using the CD-HIT-EST program (Li and Godzik 2006) (https://github.com/weizhongli/cdhit; last accessed July 15, 2020) with the parameter: −*c* = 0.95 and −*n* = 9. Considering most of SV-related TE belong to PRE1-SS (PRE-Cluster1), PRE0-SS (PRE-Cluster1), and L1-SS (L1-Cluster3), and the high variability on the terminal poly-A tail of TE, we selected PRE1a (representative sequence of PRE-Cluster1, nucleic acid positions from 1 to 231 bp) and L1-SS (representative sequence of L1-Cluster3, nucleic acid positions from 4034 to 6745 bp) as the consensus sequences for the subfamily classification of SV-related TEs. Then, we conducted subfamily classification of PRE-Cluster1 and L1-Cluster3 for all SINEs (*n* = 1,044,557) and LINEs (*n* = 1,124,441) based on the employment of significant cosegregating mutations using the COSEG software (http://www.repeatmasker.org; last accessed July 15, 2020).

In the divergence time estimation, sequence divergence of PRE and L1 from the consensus sequences (Repbase update) was obtained from RepeatMasker using the Kimura two-parameter model to predict the TE activity periods. In previous studies (Liu et al. 2009), the divergence levels were calculated and corrected for the CpG content of each repeat by *D*_CpG_ = *D*/(1 + 9*F*_CpG_) during the RepeatMasker run. The activity periods were estimated by assuming a substitution rate of 2.5 × 10^−8^ substitutions/site per generation and a generation time of 5 years (Groenen 2016). Distribution of histograms for sequence divergence of PRE and L1 was plotted using a 0.01 bin size.

### Genome Annotation of SVs

All SVs were processed by region-based or gene-based annotations using ANNOVAR software (Wang et al. 2010). There were two types of functional regions for the Duroc genome used for SV annotation, including 26,941 PCGs that were downloaded from the NCBI database (*S. scrofa* 11.1), and 28,797 LncRNAs that were identified in a previous study (Zhao et al. 2018) and are publicly available in the NONCODE database (http://www.noncode.org/; last accessed July 15, 2020). Because of the construction of LncRNA annotation was based on the Duroc genome (*S. scrofa* 10.2), we transferred the LncRNA annotation from the previous Duroc genome (*S. scrofa* 10.2) to the Duroc genome (*S. scrofa* 11.1) using the lift over pipeline (https://github.com/wurmlab/flo; last accessed July 15, 2020).

### Validation of the Three Exon-Related SVs by DNA Sequencing

Three specific SVs within exon regions were selected for technical validation by polymerase chain reaction (PCR). The primers used in the PCRs were designed in Primer5 (http://www.premierbiosoft.com/primerdesign; last accessed July 15, 2020) for amplifying the selected SV breakpoints (supplementary table S8, Supplementary Material online). The primer pairs were ∼200-bp long for all SVs and the PCRs were run using genomic DNA templates from corresponding sample genomes. Amplicons were inserted into pMD18T plasmid for DNA sequencing. After sequencing and alignment, if PCR yielded products of expected sizes and location, SVs were considered successfully validated. We designed more than two pairs of primers for each target SV locus.

## Results

### Confirmation of Two Specific Patterns by High-Confidence SVs

We applied the assembly-based SV discovery method (Li et al. 2011) to identify the high-confidence SVs from 13 pig genome assemblies using Duroc (*S. scrofa* 11.1) as the reference. All pig genome assemblies were publicly available in the NCBI databases. These assemblies included six Asian breeds, six European breeds, and Ellegaard Gottingen minipig (a crossbreed with 59% Vietnamese Potbelly Pig, 33% Minnesota Miniature, and 8% White German Landrace), which was closer to the Asian-South breed (supplementary table S1, Supplementary Material online).

We identified 13,310,147 nonredundant SVs (6,675,792 deletions and 6,634,355 insertions) with the effective length ranging from 1 to 10,000 bp (supplementary fig. S2, Supplementary Material online). As shown in the frequency per SV size (fig. 1*a*), we clearly observed two significantly SV-enriched peaks around 300 bp in length on both insertion and deletion, which contained hundreds of putative SINEs (Li et al. 2017). Comparison of SVs on different pig breeds revealed that Asian breeds had 894,030 (10.50 Mb) more SVs per genome assembly than European breeds (fig. 1*b*). The most SVs were detected in the Tibetan pig genome (2,977,640 SVs with 35.06 Mb) and the least in the Duroc pig genome (*S. scrofa* 10.2; 1,763,481 SVs with 11.40 Mb).

We further extracted four types of SV (SA-a, A-a, SA-p, and A-p types) from all detected SVs to confirm two specific SV patterns (pattern A and SA) in Eurasian species of pig (fig. 1*c*). We found 77,762 SVs in pattern SA and 88,209 SVs in pattern A, of which 17.5% and 38.7% were respectively harbored in chromosome X with the enrichment significances (*P *<* *2.1 × 10^−306^, *χ*^2^ = 5,928 and *P *<* *2.1 × 10^−306^, *χ*^2^ = 29,045) (supplementary fig. S3, Supplementary Material online). Meanwhile, a scatter plot was used to show the relationship between the size and genomic location for 47,808 specific SVs located on the X chromosome (fig. 1*d*). We observed the obvious characteristic of pattern SA that was a significant enrichment of SVs (included SA-a and SA-p types, especially to 88 SVs that >100 bp) on the 12-Mb (45–57 Mb) genomic region of X chromosome. And this 12-Mb region was exactly overlapped by the porcine 14-Mb southern haplotype in previous study (Ai et al. 2015). Similarly, the characteristics of pattern A represented a significant enrichment of SVs (included A-a and A-p types, especially to 239 SVs that >100 bp) on the 30-Mb (57–87 Mb) genomic region of X chromosome, which supported the 35-Mb SV hotspot (the SVs were clearly separated between European and Asian pig breeds) we found previously (Zhao et al. 2016).

### Verification of 327 Specific Pattern-Related SVs by Large-Scale NGS Data

In total, there were 327 SVs that showed specific patterns and their size was >100 bp. To confirm the universality of these specific pattern-related SVs, we performed a local alignment strategy (fig. 2*a*) to validate 327 SVs (supplementary table S3, Supplementary Material online) in specific patterns using large-scale NGS data. The NGS samples herein included 316 individuals from 65 different pig breeds, comprising 182 individuals from Asian breeds, 125 individuals from European breeds, seven individuals from South-East Asia, one individual from Central America, and one individual from Africa (fig. 2*b*).

As shown in figure 2*c*, we successfully confirmed two specific SV patterns in the X chromosome with an average concordance rate of 82.4% (72.8% for pattern A and 85.9% for pattern SA) (supplementary table S4, Supplementary Material online). Interestingly, we observed an apparently opposite feature in pattern A where the wild boars from Japan and North China (population 4) exhibited characteristics of European pig breeds, which was consistent with a previous SNP-based study (Ai et al. 2015). The results showed that there may be potential for gene flow between European pigs to Northern Asian wild boars.

Given the polymorphism of SV patterns in different pig breeds, we further divided all 316 pig individuals investigated into 13 main branches according to their phylogenetic relationships and divergence time (Frantz et al. 2015; Groenen 2016). These branches included *Phacochoerus africanus* (the common warthog from Africa that diverged around 11 Ma), four species of the genus *Sus* (*Sus barbatus*, *Sus verrucosus*, *Sus cebifrons*, and *Sus celebensis* that diverged around 3.5 Ma), *Sumatra* (a subspecies of *S. scrofa* that diverged around 2 Ma), and seven Eurasian pig breeds (Southern Chinese wild boar, Southern Chinese domestic pig, Tibetan wild boar, Northern Chinese domestic pig, Northern Chinese wild boar, European domestic pig, and European wild boar) (fig. 2*d*). Considering that SVs shared among different individuals were usually due to the identity-by-descent or gene flow, it was not hard to speculate that pattern A was formed after the divergence between European and Asian pigs (1 Ma) and before the divergence between South Asian and North Asian pigs (0.6 Ma). Similarly, pattern SA was formed after the divergence between South Asian and North Asian pigs (0.6 Ma) and before the divergence between Tibetan and North Asian pigs (0.2 Ma).

### The Formation of 327 Specific Pattern-Related SVs

To explore the formation of 327 specific pattern-related SVs, we first extracted the sequences of their R3 region (fig. 2*a*) based on their breakpoints (supplementary table S5, Supplementary Material online), and then clustered these sequences by multiple sequence alignment algorithm. The results showed that most pattern-related sequences had high homology with others (supplementary figs. S4 and S5, Supplementary Material online). We also observed that most of them showed 3′-terminal poly (A) tails, indicating that these sequences could be members of specific TE families (Kazazian 2004).

**Fig. 3:**
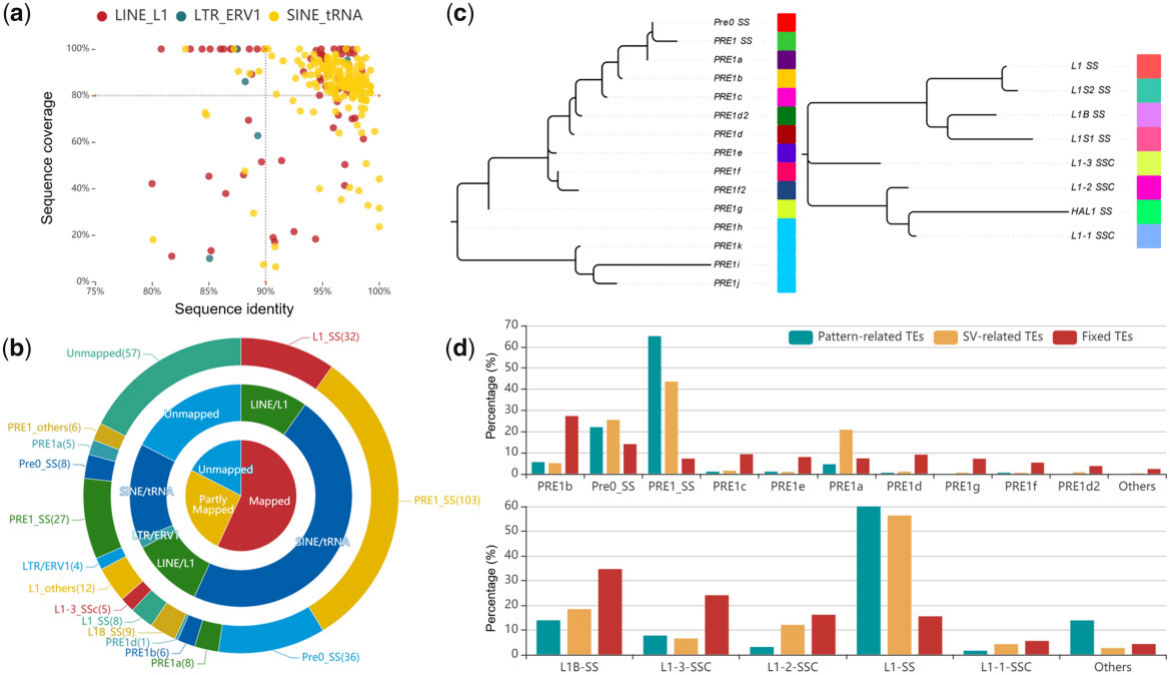
Formation of specific pattern-related SVs. (*a*) The coordinates indicate the confidence between SVs and TEs, where the *x* axis and *y* axis represent the percentage of sequence identity and sequence coverage, respectively. (*b*) The circular pie chart displays the classification and proportion of haplotype-related TEs based on how the homology is related to known TEs. (*c*) Phylogenetic tree for known PRE and L1 families in the pig genome, where different colors represent different TE subfamilies. (*d*) Bar charts indicate the percentage of SINE and LINE subfamilies from the different states, respectively. The upper bar plot shows the SINE family and the lower bar chart indicates the LINE family. The *x* axis and *y* axis represent the type of different TE subfamilies and their proportion, respectively.

Therefore, we aligned these sequences against all TEs in the pig genome and collected the best matches (fig. 3*a*). As expected, 270 sequences (82.6%) were successfully mapped to the known TE family members with high identity (>80%), of which 186 sequences were classified as complete TEs (>90% sequence identity and >80% coverage criteria), and 84 sequences were classified as partial TEs that were usually associated with SINE- or LINE-mediated retrotransposition events (Richardson et al. 2015). As shown in figure 3*b*, we further divided these complete TEs into 154 SINEs (mainly 103 PRE1-SS and 36 PRE0-SS) and 32 LINEs (32 L1-SS), indicating that the formation of pattern-related SVs was mainly due to activity of PRE1-SS (55.4%), followed by PRE0-SS (19.4%) and L1-SS (17.2%) subfamily.

To understand the phylogenetic characterization of pattern-related TEs, we constructed phylogenetic trees (fig. 3*c*) and haplotype networks (supplementary figs. S6 and S7, Supplementary Material online) based on all known members of PRE and L1 subfamilies. The results indicated that pattern-related TE subfamilies (PRE1-SS, PRE0-SS, and L1-SS) appeared to have diverged relatively late compared with other members of the same family. Interestingly, further comparing the proportion of these TE subfamilies in different states (fig. 3*d*), we found that pattern-related TEs appeared much higher proportion of PRE-1SS and L1-SS than fixed TEs (nonpolymorphic TEs). Especially, the TE-related SVs (polymorphic TEs exist in Eurasian pig breeds in the form of SVs) still have a higher proportion of PRE-1SS and L1-SS than fixed TEs, suggesting that PRE-1SS and L1-SS subfamilies were active in modern pigs.

### Activity Periods of Pattern-Related TE Subfamilies

**Fig. 4:**
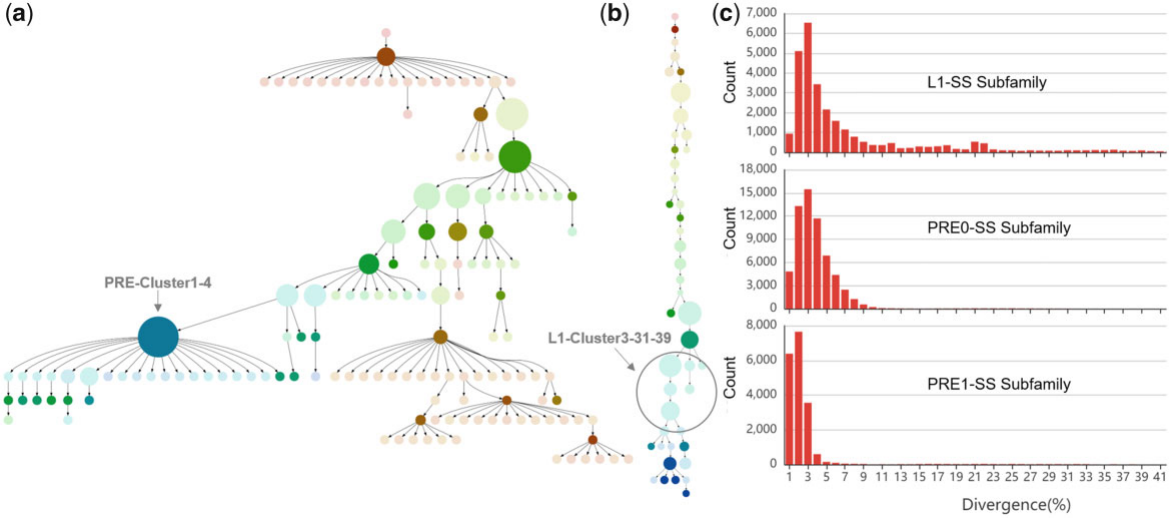
Classification and age divergence analyses of pattern-related TEs. (*a*) A MS tree of pig PRE-Cluster1 subfamilies. Each point represents a subfamily of PRE-Cluster1, and the size of the points indicates the number of corresponding subfamilies. (*b*) A MS tree of pig L1-Cluster3 subfamilies. Each point represents a subfamily of L1-Cluster3, and the size of the points indicates the number of corresponding subfamilies. (*c*) Sequence divergence distribution within the PRE1-SS, PRE0-SS, and L1-SS subfamilies. Sequence divergence distributions are plotted in bins of 0.01 increments. The activity time was calculated and displayed for PRE1-SS, PRE0-SS, and L1-SS subfamilies.

We used a distinct approach to study pattern-related TE subfamilies to determine their potential activity periods. First, we categorized the PRE1-SS, PRE0-SS, and L1-SS subfamilies from whole pig genome using the COSEG software. Based on 274,991 SINEs form PRE-Cluster1 (supplementary fig. S8, Supplementary Material online) and 4,011 LINEs form L1-Cluster3 (supplementary fig. S9, Supplementary Material online), we identified 145 distinct PRE-Cluster1 subfamilies and 45 distinct L1-Cluster3 subfamilies: The PRE-Cluster1 subfamily composition ranged from 30 to 24,292 (*P* value for subfamily partition ranged from 3e^−12355^ to 2e^−4^), and the L1-Cluster3 subfamily composition ranged from 31 to 418 (*P* value for subfamily partition ranged from 2e^−3327^ to 5e^−6^). Then, two minimum spanning (MS) trees were used to exhibit the subfamily relationships of PRE-Cluster1 (fig. 4*a*) and L1-Cluster3 (fig. 4*b*). We locally aligned the pattern-related TEs to the subfamilies using BLAST version 2.2.31+ with default parameters, we found that almost all pattern-related TEs belonged to the members of the PRE-Cluster1-4 (sub4) and L1-Cluster3-31–39 (sub31 to sub39) subfamilies (supplementary table S3, Supplementary Material online). These suggest that pattern-related TEs from the same subfamily had lower sequence divergence and their activity periods were similar.

Therefore, to further speculate the formation time of pattern-related TEs, we performed the divergence age analysis for PRE1-SS, PRE0-SS, and L1-SS subfamilies based on all their members of whole genome. We calculated the divergence level and corrected it by the CpG content of each consensus sequence using RepeatMasker. As shown in figure 4*c*, we observed the peak with small divergence in pattern-related TEs especially the PRE1-SS subfamily, implying a potential burst of pattern-related TE amplification (represented by PRE1-SS) during modern pig evolution. Further, we found that part of PRE1-SS (having the best match with pattern-related PRE1-SS; >90% sequence identity; and >90% coverage criteria) experienced the most recent activity from 0.004 to 0.025 substitutions/site. This could be estimated to have occurred from 0.08 to 4.98 Ma, assuming a substitution rate of 2.5 × 10^−8^ substitutions/site/generation and a generation time of 5 years.

### Functional Impact of Pattern-Related SVs on Pig Genome

**Fig. 5:**
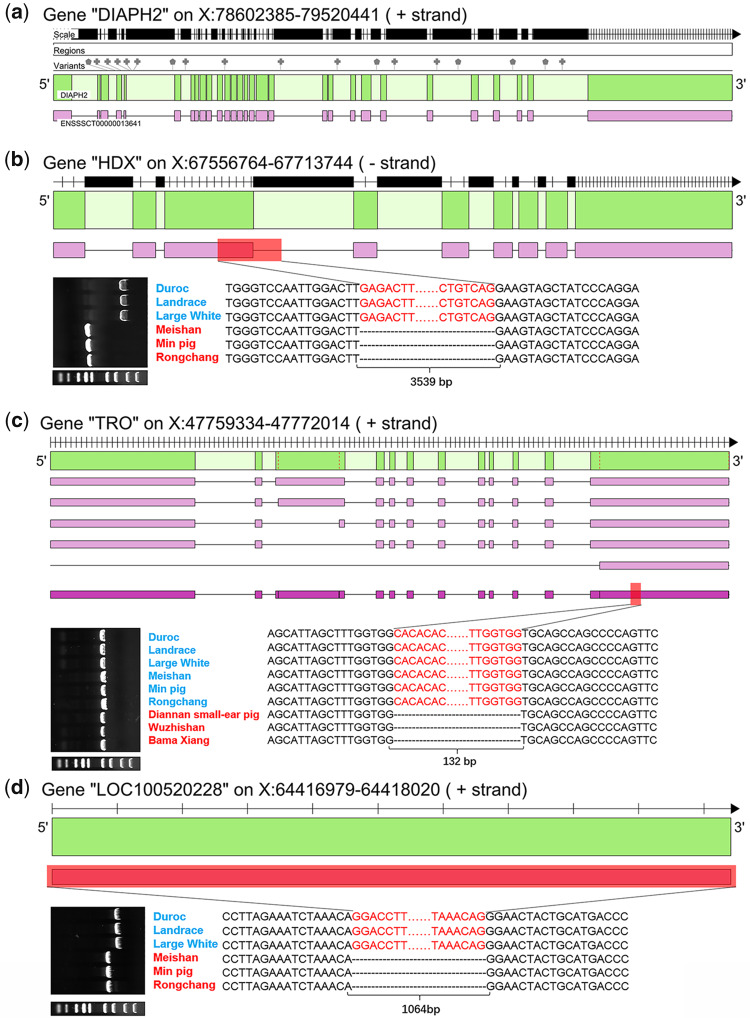
Annotation of haplotype-related TEs and classification of haplogroups. (*a*) The *DIAPH2* gene has 18 intronic TEs. The variants bar displays the 18 intronic TEs and the green and purple bars illustrate gene and transcript structure, respectively. (*b*) The HDX gene with a 3,539-bp deletion. The upper part of the diagram shows the HDX gene structure, in which green, purple, and red bars illustrate gene structure, transcript structure, and deletion variation, respectively. The bottom part of the diagram shows the PCR and sequencing results for the HDX gene within six different breeds of pigs. (*c*) The *TRO* gene with a 132-bp deletion. The content description is the same as the *HDX* gene. (*d*) The LOC100520228 gene (*SMIM1* gene) with a 1,064-bp deletion. The content description is the same as the *HDX* gene.

To evaluate the functional impacts of pattern-related SVs on the pig genome, we processed the region-based annotations for all pattern-related SVs using ANNOVAR software (Wang et al. 2010). The functional regions used for annotation included 26,941 PCG loci from the NCBI database and 28,797 LncRNA loci from the NONCODE database. The results showed that a total of 128 pattern-related SVs (39.14%) were potentially associated with 57 PCG and 19 LncRNA loci, of which 115 were in the introns of PCG or LncRNA loci (supplementary tables S6 and S7, Supplementary Material online). We found 21 PCG and 6 LncRNA loci were influenced by at least two SVs, of which the *DIAPH2* gene contained 18 pattern-related SVs (fig. 5*a*). Seven of these PCGs (*ATRX*, *BRWD3*, *CHM*, *HUWE1*, *IL1RAPL2*, *OPHN1*, and *PCDH11X*) were associated with the development of the nervous system (Ferrante et al. 2001; Philip et al. 2003; Field et al. 2007; Carrasquillo et al. 2009; Gordiyenko et al. 2010; Baptista et al. 2012; Tamming et al. 2017); *IGBP1* was involved in the regulation of the immune system (Pleiman et al. 1994); and three candidate genes (*DACH2*, *DIAPH2*, and *HDX*) played an important role in maintaining normal function of the ovary (Bione et al. 2004; Okten et al. 2013).

Importantly, the exon region of three genes was found to be altered by pattern-related SVs that were confirmed by PCR and Sanger sequencing: 1) the Highly Divergent Homeobox (HDX) gene (SINE-mediated 3,539-bp deletion in exon 3 of *HDX*, fig. 5*b*) played an important role in the development of ovarian follicles and regulation of ovarian gene expression (Okten et al. 2013); 2) the Trophinin (*TRO*) gene (132-bp deletion in exon 12 of *TRO*, fig. 5*c*) was strongly expressed in endometrial epithelial and trophectoderm cells, playing a central role in embryo implantation (Fukuda et al. 1995); and 3) the LOC100520228 gene (1,064-bp deletion over entire gene, fig. 5*d*) had orthologous gene pairs with the small integral membrane protein 1 (*SMIM1*) gene in humans, which underlies the Vel blood group and has strong effects on red blood cell traits (Cvejic et al. 2013).

## Discussion

The genome-wide detection of SVs in 13 pig genome assemblies revealed at least 0.43% genomic size difference between Asian and European pig breeds, consistent with our previous study (Zhao et al. 2016). The distribution of their size and genomic location confirmed the SV hotspot found in our previous study (Zhao et al. 2016). Further verification results based on large-scale data showed that pattern-related SVs were negligible in the *Phacochoerus africanus* and *Sus* sp. genomes (including *Sumatra*) but were present in the genomes of Eurasian domestic pigs and wild boars; this demonstrated that these pattern-related SVs were not derived from ancient substructures or unidirectional ancient interspecies introgression. Instead, they were likely derived from the independent genetic drift after the divergence between Asian and European pig breeds that included both purifying (remove deleterious TE insertions) and positive selection (favor adaptive TE insertions). Based on our findings, pattern A formation occurred from 1 to 0.6 Ma (fig. 2*c*), which corresponded to the early Middle Pleistocene, after the divergence between Asian and European pig breeds (Groenen et al. 2012; Frantz et al. 2013) but before the divergence between Southern and Northern Chinese pigs. The pattern SA likely appeared between 0.6 and 0.2 Ma in the late Middle Pleistocene (fig. 2*c*), after the divergence between Southern and Northern Chinese pigs but before the divergence between Tibetan wild boars and Northern Chinese pigs.

Further exploration of the content of the pattern-related SVs showed a strong association between the historical expansion of TE families and formation of pattern A and SA. For example, the classification and divergence age analysis for PRE1-SS subfamily indicated an amplification of the PRE-Cluster1-4 subfamily during the divergence of modern pigs. Most TEs were neutral or even deleterious to organisms and hard to spread in the whole population (Baduel et al. 2019; Carpentier et al. 2019); however, they can also be potentially recruited in adaptive processes and rise in frequency through the entire population due to positive selection (Bourgeois and Boissinot 2019). Therefore, we speculate that these pattern-related TEs belong to adaptive haplotypes (either due to an adaptive TE or hitchhiking with a nearby adaptive polymorphism) that were related to distinct biological functions. These biological functions were probably related to the development of the neuron, immune system, thermoregulatory system, and maternal reproductive systems. However, the relationship between these traits and pattern-related TEs and the functional roles of three pattern-related genes (*HDX*, *TRO*, and *SMIM1*) need further investigation at the molecular level.

**Fig. 6:**
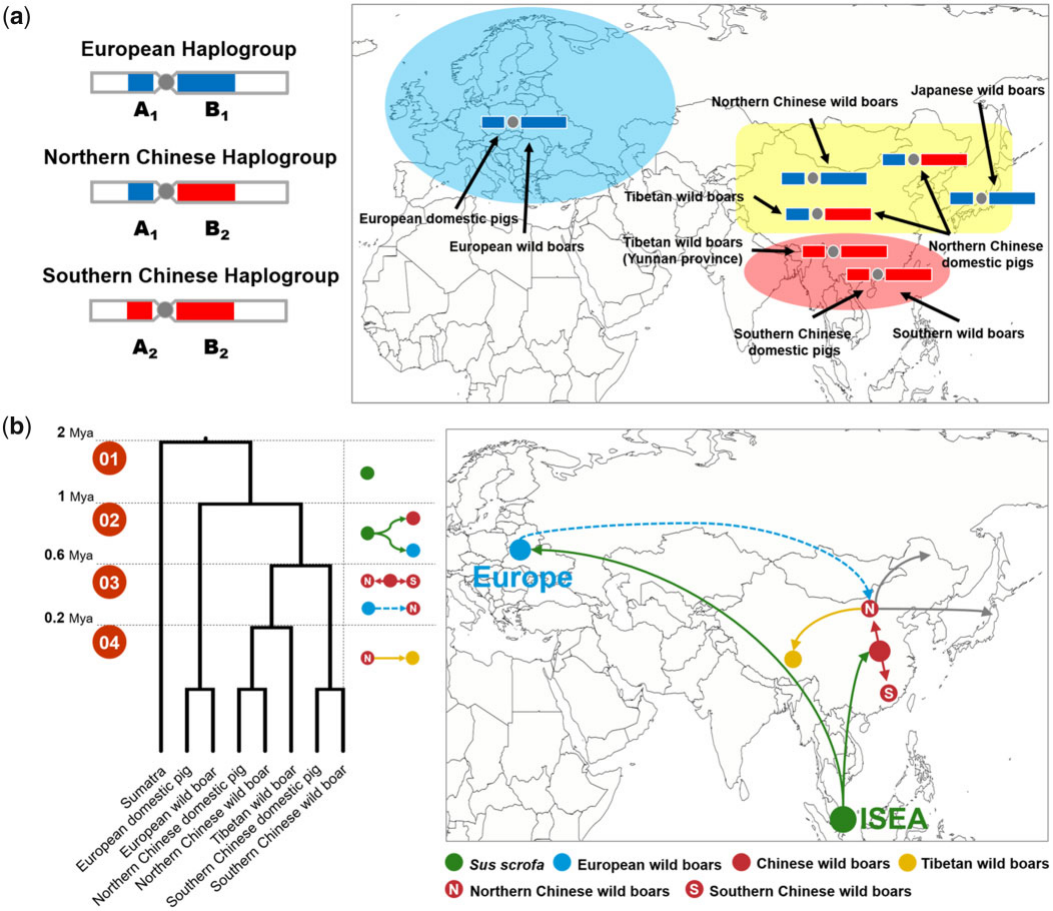
Overview of Asian-Western structural variation and its formation. (*a*) Geographic distribution of three specific haplogroups. The three haplogroups represented in the modern pig genome across Eurasia. The blue, yellow, and red shadings represent Europe, North Asia, and South Asia, respectively. (*b*) Four periods characterizing the formation of distinct haplogroups during the evolutionary history of Eurasian pigs. Each point represents a population of pigs. Each line represents the route and direction of the migration of each population. The solid lines represent the known evolutionary history, and the dotted lines represent the novel evolutionary events inferred in this study.

It is worth discussing why the same TE subfamilies with the same active period eventually lead to two specific patterns, and the regions of the two specific patterns were closely adjacent and separated by a centromere. As shown in previous studies, the “centromere effect” was able to reduce the likelihood of recombination and generate the recombination desert that eventually leads to homozygous regions in the genome (Pozzoli et al. 2008; Rubin et al. 2012; Wang et al. 2015). This genomic structural feature ensured that pattern-related TEs stayed within the region of the whole pattern (45–87 Mb), resulting in the significant structural difference between Asian and European pig breeds. From our current findings, pattern SA with 12 Mb and pattern A with 30 Mb can be integrated as the three haplogroups that were observed in the modern pigs (fig. 6*a*). It was clear that pattern A was formed during the independent evolution of Asian and European pigs between 0.6 and 1 Ma (Groenen 2016). However, there was still no evidence supporting the formation of pattern SA, although some hypotheses have been put forward. One hypothesis for pattern SA formation suggested an admixture event between Southern Chinese wild boars and an extinct species (Groenen 2016); however, our findings suggest no obvious difference between pattern SA- and pattern A-related TEs, indicating that they were simultaneously formed between 0.6 and 1 Ma. Additionally, we observed that the pattern SA-related TEs were not present in other *Sus* species from Island South-East Asia, suggesting little association between the pattern SA and the gene flow from other *Sus* species. Another hypothesis for pattern SA was that the individuals who carried A_1_ haplotypes (pattern SA, fig. 6*a*) had an advantage in northern latitudes, and therefore became fixed within northern Asian pigs and was subsequently transmitted to European breeds by migration across Eurasia (Ai et al. 2015). This hypothesis provided a conservative explanation for why A_1_ haplotypes exist only in the North Asian, but it ignored the fact that Northern Chinese wild boars also carried the B_1_ haplotype (fig. 6*a*), which was more likely derived from European pigs. More importantly, it was hard to explain why the B_2_ haplotype (fig. 6*a*) disappeared in European populations. Therefore, further combining our findings, we put forward a more conservative hypothesis that the pattern SA and A were simultaneously formed during 0.6 and 1 Ma, and European pigs with A_1_B_1_ was subsequently transmitted to North Asian by migration due to the reduction in global temperature. European and North Asian hybrid pigs who carry the A_1_ genotype are more easily adapted to northern latitudes, resulting in the two specific patterns we identified here.

By combining the previous hypothesis and our current findings, we were able to make a more conservative guess for the formation of pattern A and SA during the Middle Pleistocene. As shown in figure 6*b*, we defined four phases to characterize the changes of these distinct patterns during the evolutionary history of Eurasian pigs. Phase 1: around 1 Ma, there was no difference in SVs among all *Sus* species, all pattern-related TEs had not yet been formed. Phase 2: in the early Middle Pleistocene (between 1 and 0.6 Ma), the European and Asian wild boars diverged from Island South-East Asia, expanding and evolving across Eurasia. The adaptation to novel habitats and hybridization with local wild boars led to huge genomic differences. This included the minor allele frequencies at millions of genomic locations and active TE families causing abundant SVs. The low recombination provided a remarkable region of homozygosity at the center (45–87 Mb) of the X chromosome, gradually resulting in distinct patterns (A_1_B_1_ and A_2_B_2_) between Asian and European breeds (fig. 6*b*). Phase 3: in the late Middle Pleistocene (0.5–0.2 Ma), the significant reduction in global temperature contracted forests into small refugia isolating populations across Mainland Southeast Asia. There was a clear genetic divergence between South Asian (South China) and North Asian (North China, Tibet, and Japan) wild boars (Frantz et al. 2013). At the same time, European wild boars migrated to North Asia and admixture events occurred with North Asian pigs (North China, Tibet, and Japan), resulting in four recombinant haplotypes (A_1_B_1_, A_1_B_2_, A_2_B_1_, and A_2_B_2_). Low temperatures made it easier for the haplotype carrying A_1_ to survive, causing only haplotype A_1_B_1_ and A_1_B_2_ to remain in North Asian wild boars. Phase 4: around 0.2 Ma, Tibetan wild boars separated from North Asian wild boars with the A_1_B_2_ haplotype, and both A_1_B_1_ and A_1_B_2_ haplotypes remained in North Asian wild boars (North China and Japan).

In conclusion, these two specific patterns of TEs could be used to help us understand the evolution and movement of *S. scrofa* across Eurasia. Therefore, we believe that the TE families caused SVs to be unique genetic markers, providing new insights into the potential gene flow between different pig breeds.

## Supplementary Material

Supplementary data are available at *Genome Biology and Evolution* online.

## Supplementary Material

evaa142_Supplementary_Data

## Notes

**Data deposition:** In this study, 14 genome assemblies from 13 pig breeds were downloaded from NCBI (ftp://ftp.ncbi.nlm.nih.gov/genomes; last accessed July 15, 2020) for structural variant (SV) detection. The NGS data of all 316 samples have been deposited at the NCBI database under the accesion PRJNA13421, PRJNA144099, PRJNA176189, PRJNA309108, PRJNA186497, and PRJNA13421. Other relevant data are included in this article and supplementary files, Supplementary Material online.
