# Supplementary material for: PRE-1 Revealed Previous Unknown Introgression Events in Eurasian Boars during the Middle Pleistocene
Source: Genome Biol Evol. 2020 Jul 10;12(10):1751–64. doi: 10.1093/gbe/evaa142 (PMC7643367; doi:10.1093/gbe/evaa142)
Supplement: evaa142_Supplementary_Data [file evaa142_supplementary_data.zip › Supplementary Figures.pdf]

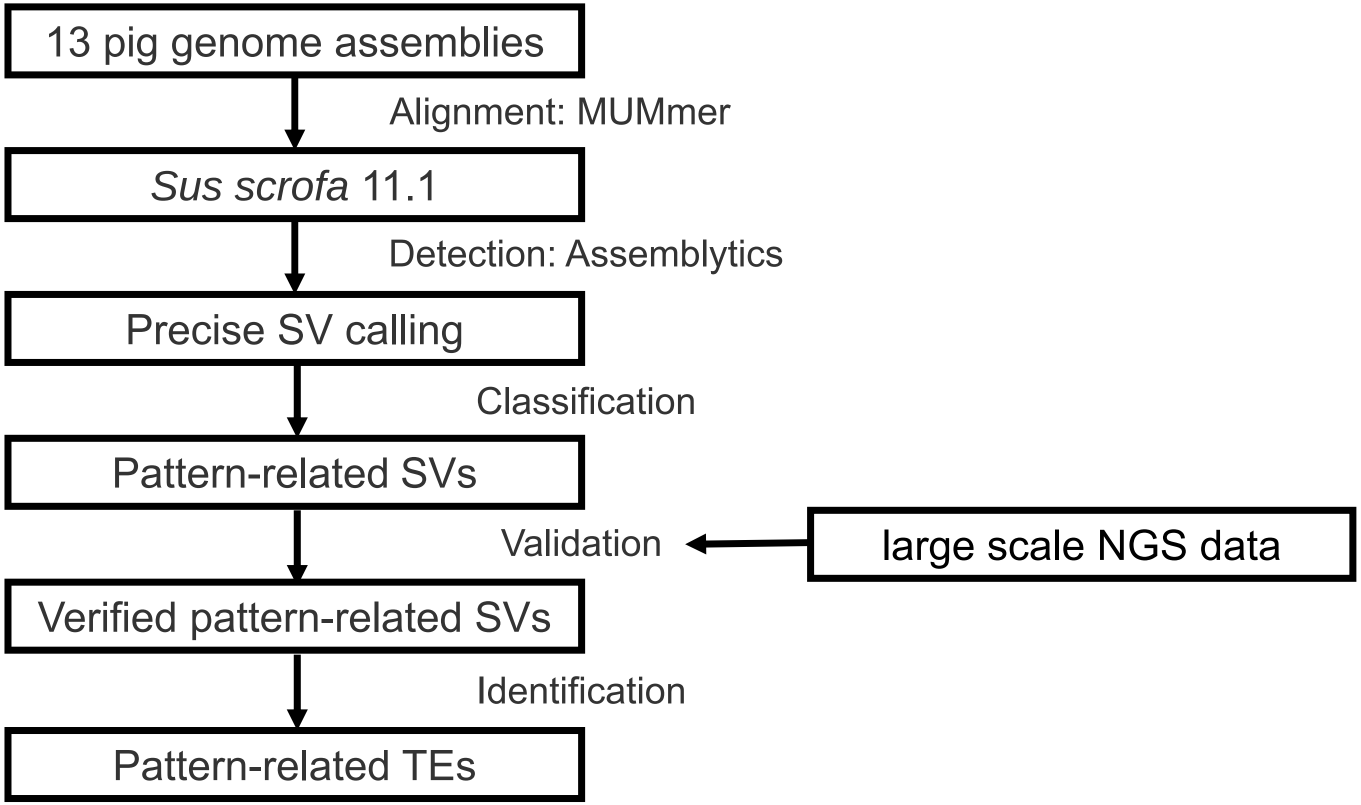

Figure S1: Pipeline of the assembly-based strategy and large scale NGS-based validation.

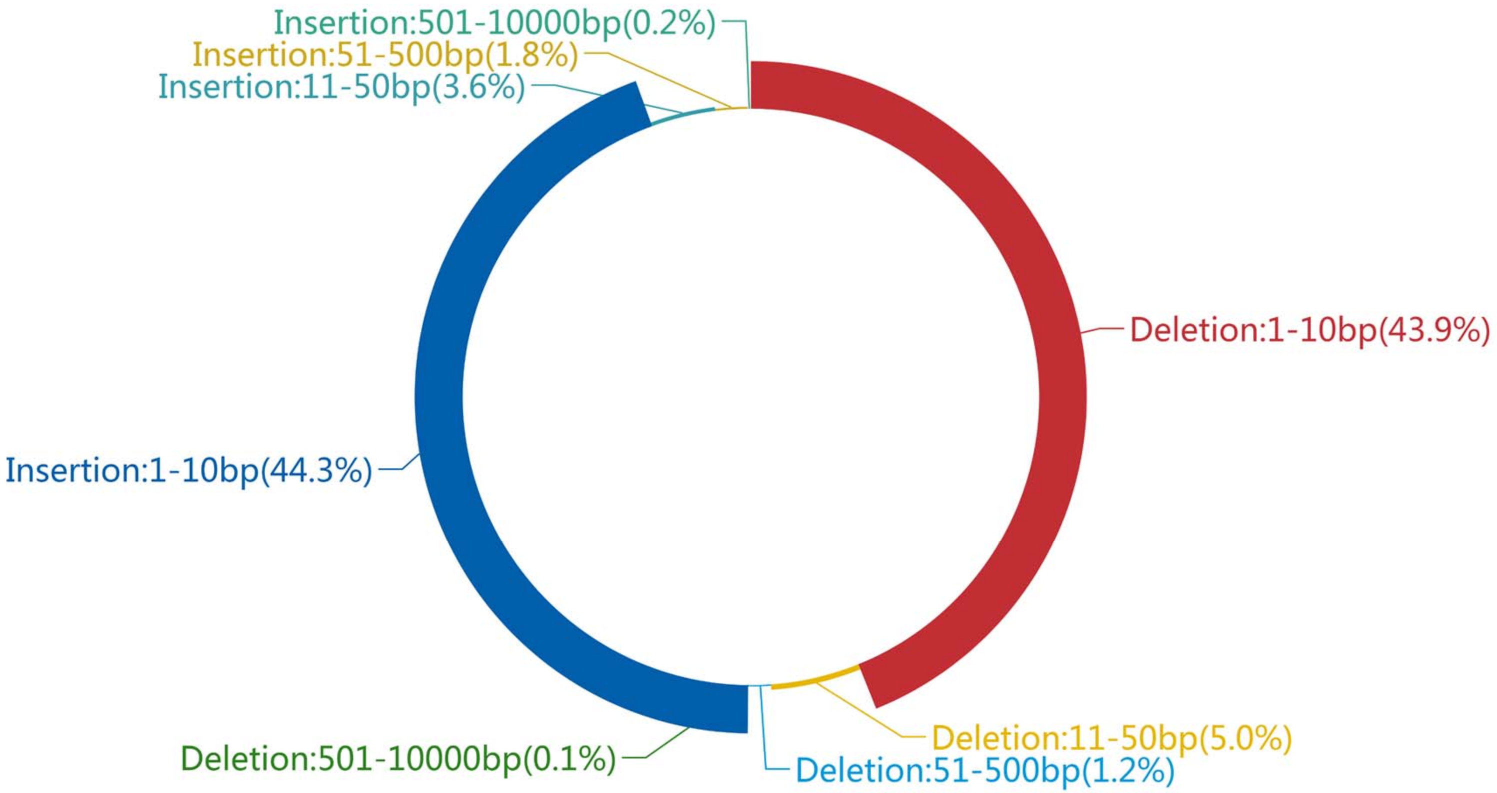

Figure S2: Pie chart for the insertions and deletions with different length ranges

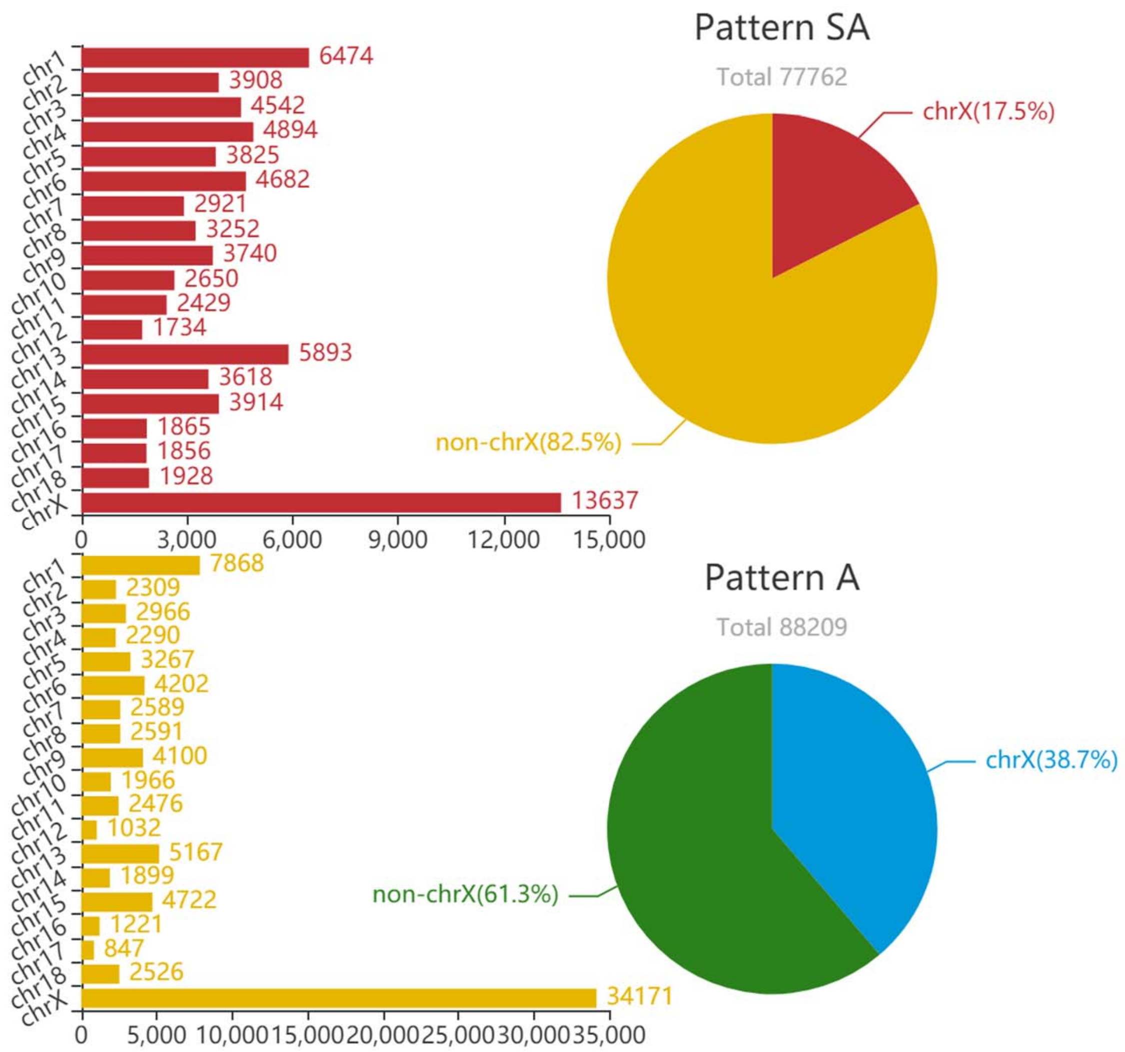

Figure S3：Chromosome distribution of pattern-related SVs from two specific patterns

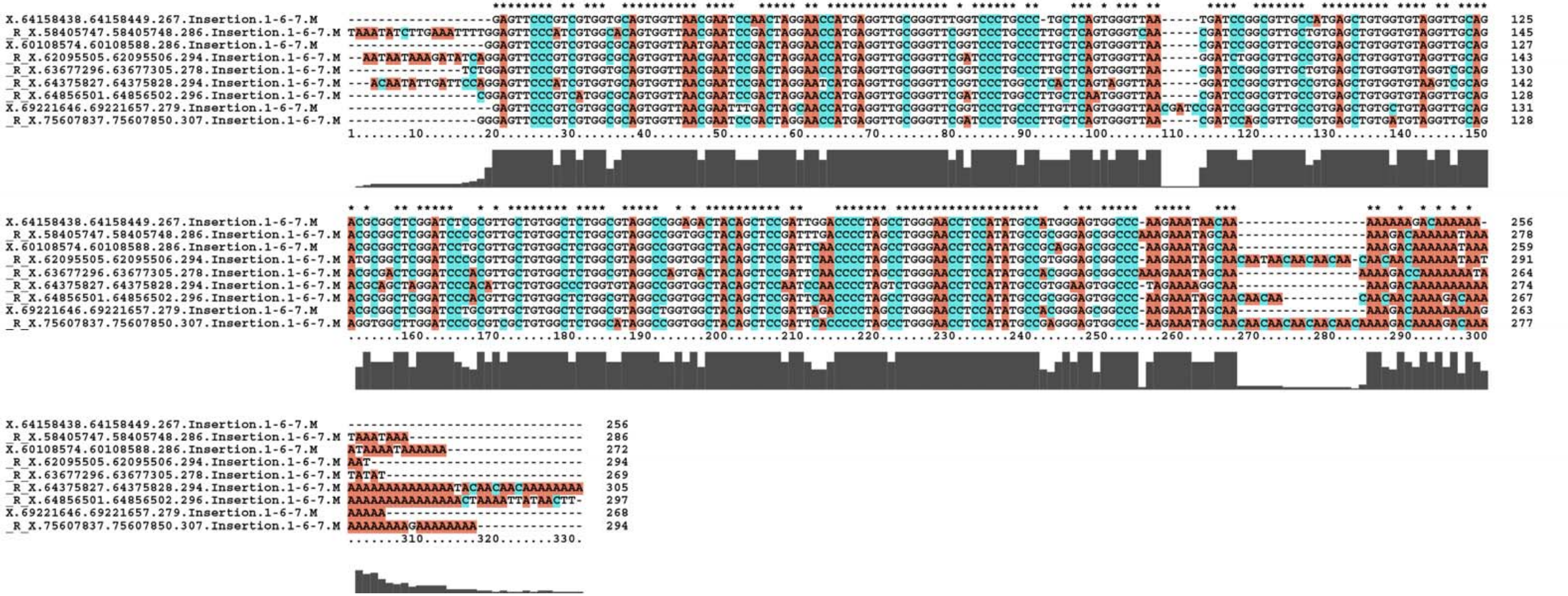

Figure S4: Multiple alignment of SINE pattern-related sequences

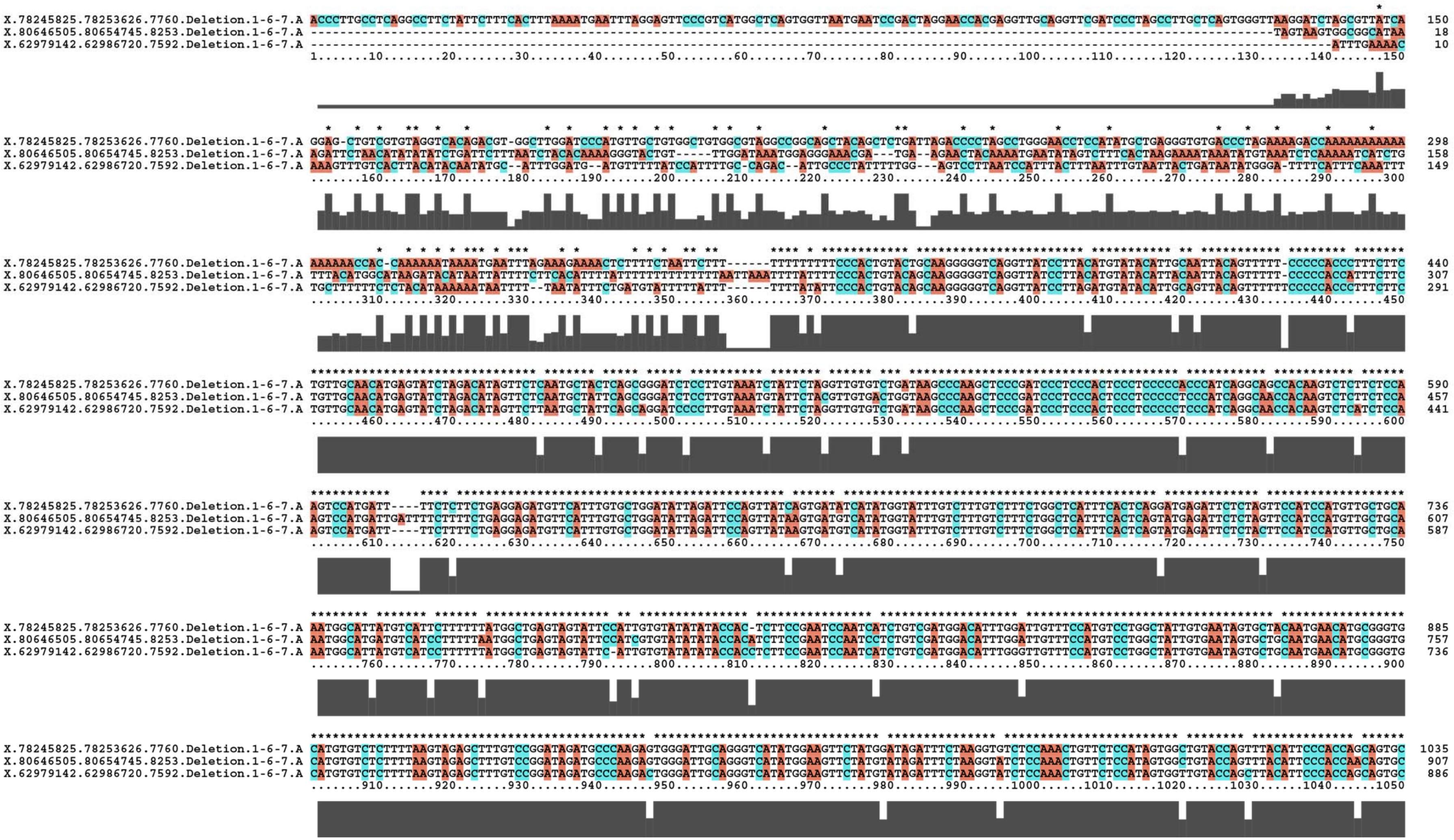

Figure S5: Multiple alignment of LINE pattern-related sequences

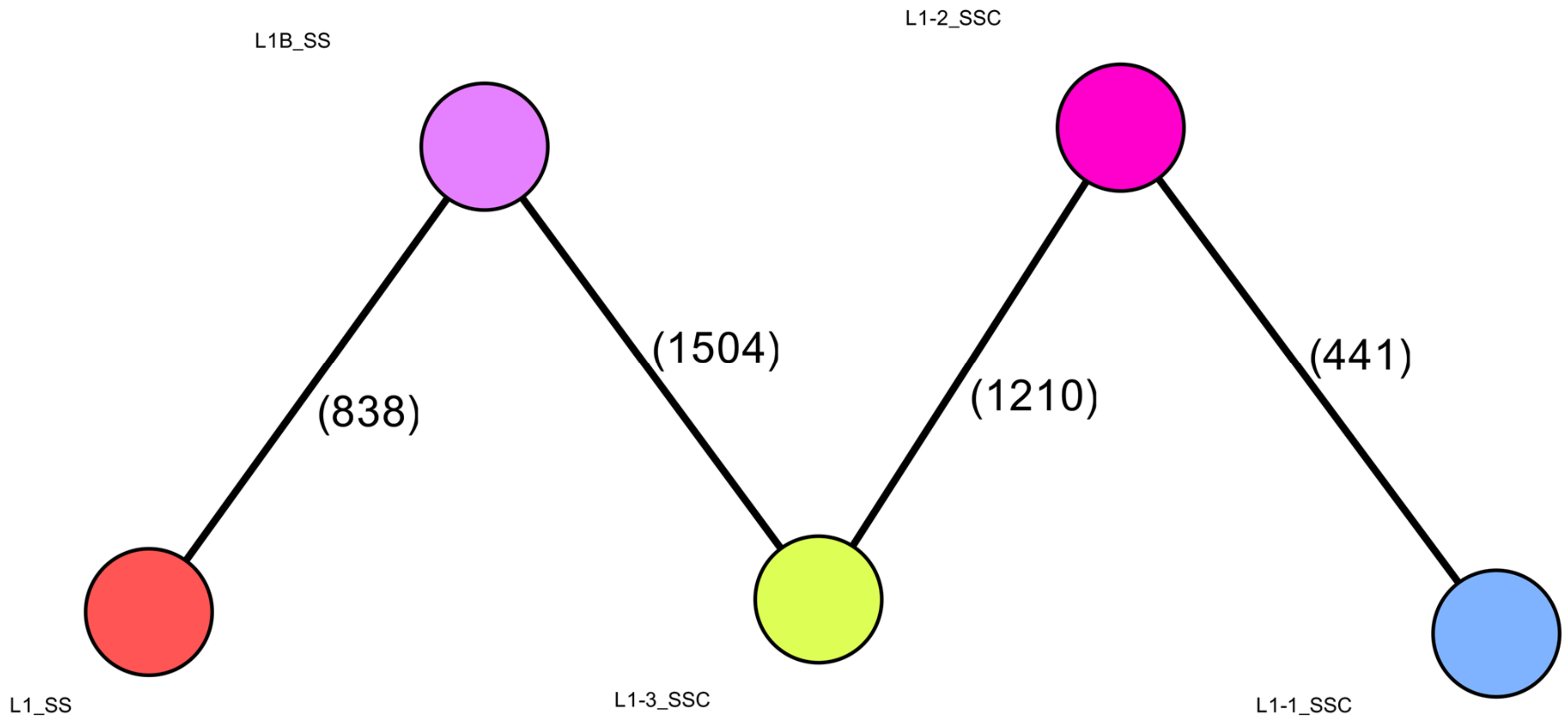

Figure S6: Haplotype network plots for known L1 subfamilies

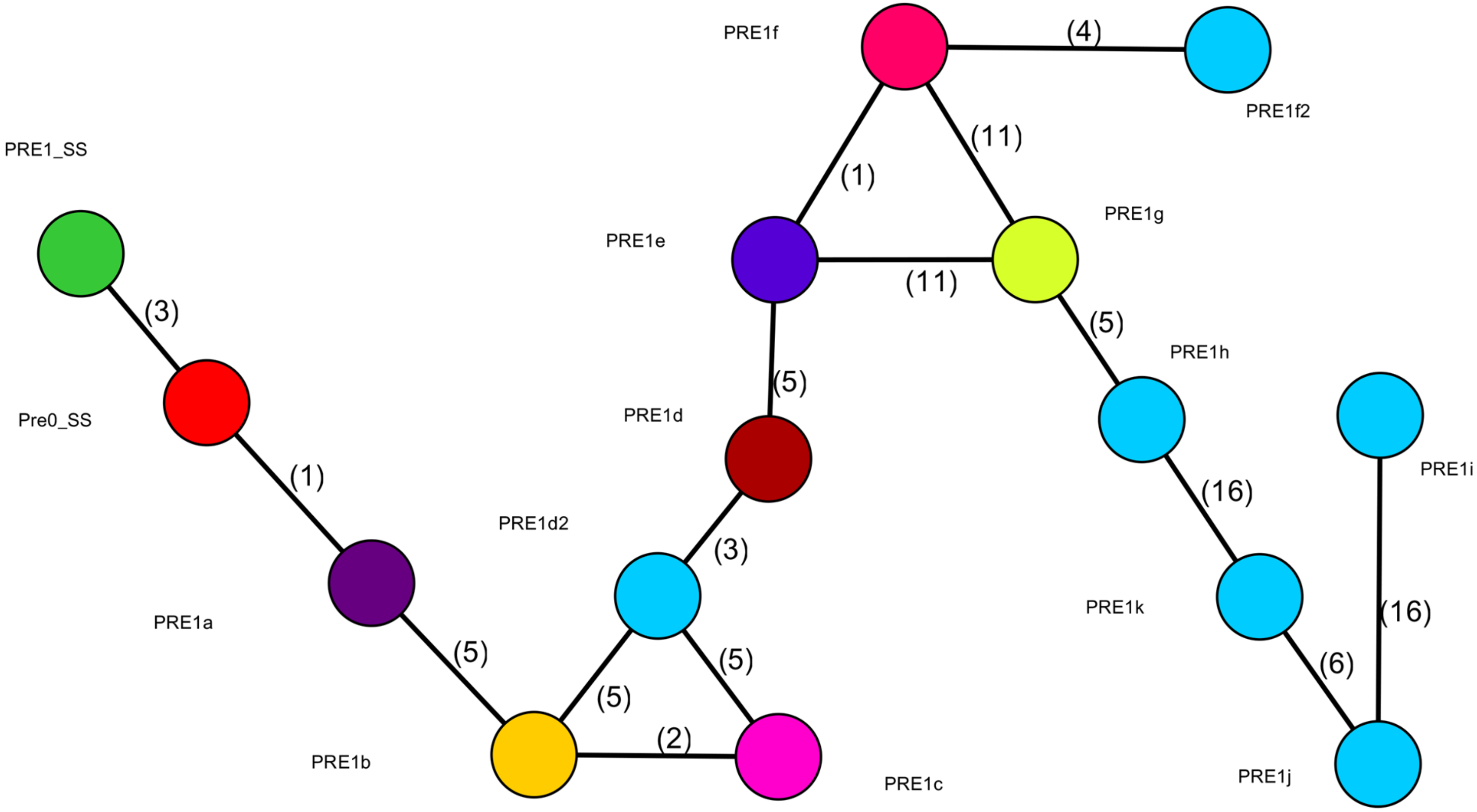

Figure S7: Haplotype network plots for known PRE subfamilies

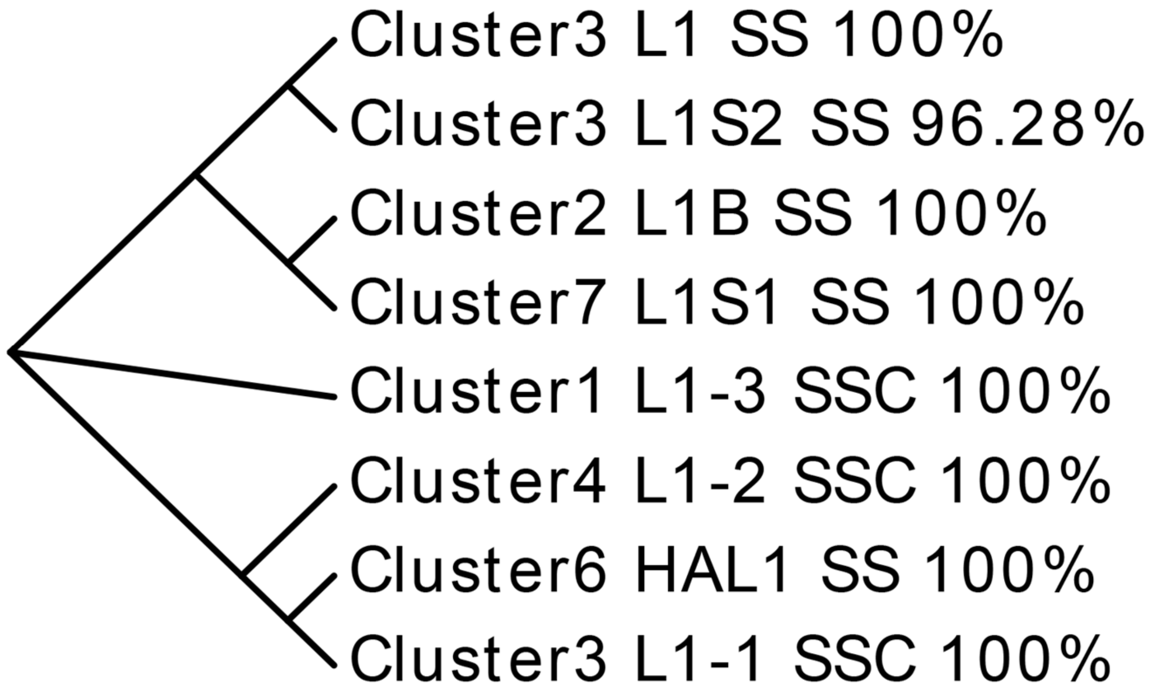

Figure S8: The phylogenetic tree for 8 known pig LINE TEs

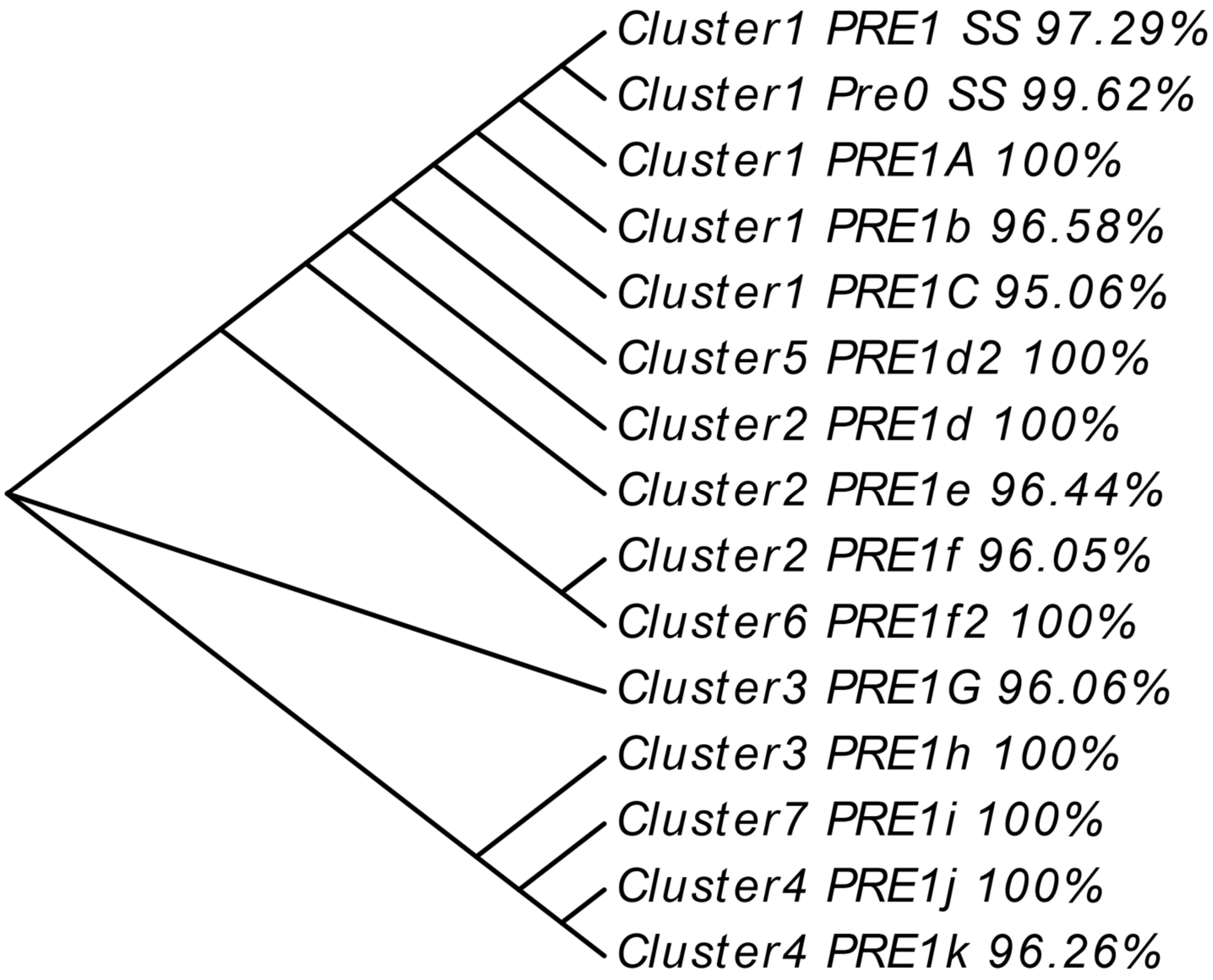

Figure S9: The phylogenetic tree for 15 known pig SINE TEs
