# Supplementary material for: PRE-1 Revealed Previous Unknown Introgression Events in Eurasian Boars during the Middle Pleistocene
Source: Genome Biol Evol. 2020 Jul 10;12(10):1751–64. doi: 10.1093/gbe/evaa142 (PMC7643367; doi:10.1093/gbe/evaa142)
Supplement: evaa142_Supplementary_Data [file evaa142_supplementary_data.zip › Response to Editors.doc]

**Ref: GBE-190934.R1**

Title: **PRE-1 Revealed Multiple Previously Unknown Introgression Events in Eurasian Boars durings the Middle Pleistocene**

Journal: Genome Biology and Evolution (**GBE**)

**Editor' Comments:**

Dear Dr Liu,

I have now received the comments from the two reviewers. As you will see, the reviewers appreciate the work you have done to answer all their previous comments. It remains some particular points to answer, that you should be able to do easily. In particular, I will insist on one point that concerns the need to improve the quality of the writing by making it read by a native English speaker using professional proofreading service. This is why I am recommending a minor revision of your manuscript, hoping the reviewer comments will help you improve your work.

Dear Editors,

I am glad to hear from you and thank you very much for your positive comments again, which have contributed this manuscript so much. we agree to all of the suggestions you have mentioned in our paper and we have incorporated all the revisions as your kind remarks presented in yellow highlights. In addition, the manuscript has already been modified by the professional editing company and it was also presented by the yellow highlights. We hope that our latest revision has satisfactorily addressed all the comments and will now be acceptable for publication in your esteemed journal, ***Genome Biology and Evolution***.

**Reviewers' Comments:**

**Referee: 1**

**Comments to the Author**

**Comment:** I have now reviewed the revised version of the manuscripts from Zhao et al., entitled "PRE-1 Revealed Previous Unknown Introgression Events in Eurasian Boars during the Middle Pleistocene". The authors did work hard to improve the quality of the manuscript and their responses to my comments where thorough and satisfactory. I think the data provided are very relevant and original and will be a great addition to the collection of articles in GBE. However, there is still some issues regarding the writing which impacts the way the authors' hypothesis and results can be interpreted.

I reported two types of comments: 1, more "major" regarding the science, methods and understanding that must be addressed upon publication. 2, then I report typos, syntax and misspelling (the ones I caught). I suggest the authors reach out to a native English speaker or a professional service to polish their manuscript before publication.

**AU:** Thank you very much for your work! As your suggestions, we have provided detailed responses to all of your comments point-by-point in the revised manuscript. And the manuscript has already been modified by the professional editing company. It is presented by the yellow highlights.

Major:

**Comment:** 1 - please list the advantage(s) of SV vs SNP for the current investigation

**AU:** Thank you for your question. At first, we need to accept that SV is only another genetic marker, not a complete substitute for SNP. However, SV still has some advantages over SNP:

1) SVs broaden our understanding of the genetic basis of evolution, diversification, and domestication. (PMID: 30745056)
2) SVs can alter the copy number of regulatory elements or modify the 3D genome by disrupting higher-order chromatin organization such as topologically associating domains (PMID: 29692413)
3) SVs are associated with complex phenotypes and SVs play a large role in rare disease biology and shaping heritable gene expression diffserences in the human population (PMID: 32460305)
4) The effect of SVs is stronger than the association of SNPs with gene expression variation (PMID: 31028401)

We hope you'll be satisfied with our answer.

**Comment:** 2 - the whole section on SV presence/absence is much better and clearer than the first version

**AU:** Thank you very much for your positive comments. We are so sorry for poorly written and badly description of SV presence/absence in our first version. We believe that we have also improved our manuscript to respond your major concerns in current revised manuscript.

**Comment:** 3 - The authors do not consider in detail the TE shared between populations but still segregating TE (figure 1): however, these markers can still provide information about the extent of genetic differentiation. Can the authors discuss the proportion of population-specific vs non population-specific polymorphic TE insertions? It is important to contrast candidate loci (high differentiation) with other loci (baseline differentiation) and the local recombination rate in order to infer selection.

**AU:** Thank you very much for your constructive suggestion. Do you mean the “population-specific” is “Asian/European-specific”? if it is “Asian/European-specific”, the proportion of “Asian/European-specific” is far lesser than “non-Asian/European-specific”. And the “Asian/European-specific” is mainly focused on X chromosome but “non-Asian/European-specific” is scattered throughout the whole genome. In fact, previous studies have shown that there is very low recombination rate (PMID: 25526890) and the clear selection signatures (PMID: 23151514) at both of pattern A and SA.

**Comment:** 4 - L. 111: randomly inserting copies of themselves --> be careful here as this is not the case of all TE families and the process cannot be directly described as random. I would replace "randomly dispersing" by "spreading out copies of [...] in the genome"

**AU:** Thank you for your kindly suggestion. We are sorry for our confused description of TE. We have revised the description for TE in our revised manuscript. New descriptions at Page 4 line 111 to 113 in our revised manuscript.

**Comment:** 5 - L. 121: " suggesting that porcine TEs likely have a similar function to human TEs". I am not certain to understand what function the authors mean here. I assume it is hypothesized that the common ancestry between the human and pig 7SL RNA SINEs suggest convergence 1) in their biochemical properties 2) the mobilization and insertion mechanisms 3) conflicts/benefits for the host.

**AU:** Thank you for your guideline, we have corrected and added your constructive suggestion in this description. New descriptions at Page 5 line 121 to 123 in our revised manuscript.

**Comment:** 6 - L. 131: I feel that "pattern" can be replaced by "haplotype" but I would like the authors to confirm.

**AU:** Thank you for your guideline, we have corrected the description in revised manuscript. New descriptions at Page 5 line 131 in our revised manuscript.

**Comment:** 7 - L. 225: how these loci were selected?

**AU:** Thank you for your question. Do you mean how we to select the “327 specific pattern-related SVs” in the L.325? We have added the new description for explaining how these 327 loci were selected at Page 11 line 301 to 302 in our revised manuscript.

**Comment:** 8 - L. 228: It is still confusing to me to read "sequence downstream the poly-A signal". If this sequence isn't a TSD, what sequence, downstream the poly-A of a SINE that makes it "specific TE families"? Or do they refer to the poly-A? This would make sense to me.

**AU:** Thank you very much for your question. Do you mean the “sequence downstream of the poly(A) signals” in L.328? We are very sorry for confusing you by our unclear description. The poly(A) signals are not the downstream region of SINE but to the 3′-terminal poly (A) tail of SINE structure. We have updated the description in our revised manuscript. New descriptions at Page 12 line 334 in our revised manuscript.

**Comment:** 9 - L. 342: "divergence" --> "have diverged". I do not understand what the authors mean by divergence here: is it divergence between copies of a given family (diversification, radiation,...) or between different families?

**AU:** Thank you for your pointing our mistake, we are very sorry for confusing you by our unclear description. The divergence here means the divergence between different subfamilies, we have corrected the description in revised manuscript. New descriptions at Page 13 line 348 in our revised manuscript.

**Comment:** 10 - L. 345: "SV-related TEs" I would say "TE-related SVs" or "TE-induced SVs" (the TE is the cause of the SV).

**AU:** Thank you very much for your constructive suggestion. We have updated the description in our revised manuscript. New descriptions at Page 13 line 351 in our revised manuscript.

**Comment:** 11 - L. 357 (review syntax: "Further blasted pattern-related TE...")

**AU:** Thank you very much for your question. We have revised the description in our revised manuscript. New descriptions at Page 13 line 364 to 369 in our revised manuscript.

**Comment:** 12 - L. 364: "and corrected it by the CpG content of each consensus sequence." --> the corrected value is calculated during the RepeatMasker run and therefore is already calculated in the RM results.

**AU:** Thank you very much for your question. We are very sorry for confusing you by our unclear description. The description that "and corrected it by the CpG content of each consensus sequence." is actually refers to the corrected value from RM results of RepeatMasker. We have revised the description in our revised manuscript. New descriptions at Page 13 line 372 to 373 in our revised manuscript.

**Comment:** 13 - L. 372: Can the authors provide a critical review of the molecular clock used? (limitation, confidence interval).

**AU:** Thank you for your suggestion. we are very sorry that we unable to provide the critical review for the molecular clock. The periods estimation in our study were mainly referred to previous study (PMID: 19411604). We appreciate your contribution to our manuscript.

**Comment:** 14 - L. 400: this difference could be measured in terms of genetic distance / differentiation. See PMID for inferences from dominant markers: 17850542; 28517033

**AU:** Thank you for your constructive suggestion and technical assistance. TE-based genetic distance / differentiation is really a greet method to reveal the genetic differentiation between populations. We will apply this idea to our ongoing TE-based study. However, we are sorry for our confusing description. In this study, the genomic difference represented the difference in genomic sequence size between Asian and European pig breeds. We have revised the description at Page 15 line 408 to 410 in our revised manuscript.

**Comment:** 15 - L. 405-407: In my view, the main evolutionary process (or at least the null hypothesis) should be genetic drift occurring after the Asian/European split; in a lesser extend negative selection (removes deleterious insertions), and, more rarely, positive selection (favors adaptive insertions) may also influence the TE insertion frequencies.

**AU:** Thank you for your pointing our mistake. As your suggestions, we have corrected the description at Page 15 line 415 to 418 in our revised manuscript.

**Comment:** 16 - L419-420: This is a rather dated reference. I do think that most TE insertions evolve neutrally > deleterious > advantageous. However, a lot of parameters can influence these results, and would be relevant to gather evidence in pigs supporting each possibility.

**AU:** Thank you very much for your pointing our mistake. As your suggestion, we also consulted some latest literatures (PMID: 31862875, 30604755), and we agree with you on the viewpoint that “neutrally > deleterious > advantageous”. Therefore, we have corrected the description at Page 16 line 428 to 429 in our revised manuscript.

**Comment:** 17 - L. 423-425: Sorry, this is not clear to me. As is, I read that the pattern-related TEs (which ones?, all of them or the 300+ selecting showing sharp differences?) is associated with different functions due to local adaptation. There is not evidence supporting this hypothesis. However, it is indeed likely that among these TE some belong to adaptive haplotypes (either due to an adaptive TE, or hitchhiking with a nearby adaptive polymorphism

**AU:** Thank you very much for your question, we are sorry for our confusing description. In our study, the pattern-related TEs mean the TEs that showed sharp differences between Asian and European pigs. Same as your suggestion, we wanted to say that these pattern-related TEs were formed because of adaptive polyTE, or hitchhiking with a nearby adaptive polyTE. We have corrected the description at Page 16 line 432 to 434 in our revised manuscript.

**Comment:** 18 - L. 482: I think that this can be done using ALL segregating TE, not only the population-specific one.

**AU:** Thank you very much for your constructive suggestion. We are very sorry for confusing you by our inaccurate description. In this study, we mainly want to verify and refine the SV hotpot regions and proposed a hypothesis to explain its formation. Just as you suggested, we’ve been working on using ALL segregating TE to reconstruct the evolution and movement of *S. scrofa* across Eurasia. We hope to be able to share these results in the future. We have revised the appropriate description at Page 18 line 490 to 491 in our revised manuscript.

**Minor, typos:**

**Comment:** 19 - Please review the time used in the sentences. Too much are using the past/preterit while present apply.

**AU:** Thank you for your guidance. We are sorry for the tense mistake in our manuscript. We have thoroughly updated the time used in revised manuscript.

-L. 94: admixture --> noun instead of participle present (admix)

**AU:** Thank you for your guideline, we have corrected the description in revised manuscript. New descriptions at Page 4 line 93 to 95 in our revised manuscript.

-L.102: there are much more recent example of the study of SV and TE-SV in humans (PMID: 21876680; 26432246; 28369037)

**AU:** Thanks a lot for your suggestion. We had added these examples in our revised manuscript.

-L.124 "of A previous study"

**AU:** Thank you for your guideline, we have added the cited paper in revised manuscript. New descriptions at Page 5 line 125 in our revised manuscript.

-L.127-128: these genome assemblies were not introduced before, please add more contect for the reader.

**AU:** Thank you for your guidance, we are sorry for our confusing description. New descriptions at Page 5 line 127 to 128 in our revised manuscript.

-L.128-129: "area": does the authors mean locus or haplotype?

**AU:** Thank you for your suggestion, we are sorry for our confusing description. New descriptions at Page 5 line 129 in our revised manuscript.

-L.129: "...SVs were segregated" --> "segregate"

**AU:** Thank you for your guideline, we have corrected the description in revised manuscript. New descriptions at Page 5 line 131 in our revised manuscript.

-L. 135:  "63.7% belonged to TE family" --> the family name is missing

**AU:** Thank you for your pointing our mistake, we have corrected the description in revised manuscript. New descriptions at Page 5 line 137 in our revised manuscript.

-L. 151 (and anywhere else): "assembly genomes" --> genome assemblies

**AU:** Thank you for your guideline, we have corrected all "assembly genomes" to "genome assemblies" in revised manuscript.

-L. 161 "a cluster is consists" -> remove "is"

**AU:** Thank you for your guidance, we are sorry for our carelessness and revised this mistake. New descriptions at Page 6 line 162 in our revised manuscript.

-L. 169: "pattern A and SA" are not described before and should be explained.

**AU:** Thank you for your guidance. We have added the expand description of “A” and “SA” when the first time they use it. New descriptions at Page 6 to 7 line 169 to 176 in our revised manuscript.

-L. 169-170: replace "our house" by "in house"

**AU:** Thank you for your guidance, we are sorry for our carelessness and revised this mistake. New descriptions at Page 6 line 175 in our revised manuscript.

-L. 174: "represents the genomic sequence is only presence" --> "represent the genomic

sequences only found in Asian pig breeds and not in European breeds"

**AU:** Thank you for your kindly suggestion. We have revised the description in our manuscript. New descriptions at Page 7 line 172 to 180 in our revised manuscript.

-L. 219: "positions": I think the authors mean "loci" (locus: singular; loci: plural)

**AU:** Thank you for your kindly suggestion. We have revised the statement in our manuscript. New descriptions at Page 8 line 224 in our revised manuscript.

-L.283: "totally": does the authors mean "We found 77k complete SV pattern"? Even though I am still not sure what is meant.

**AU:** Thank you very much for your question. We are very sorry for confusing you by our unclear description. We have removed the "totally" to avoid the confusing description.

-L. 297: Please remind here how the 327 SV were selected

**AU:** Thank you for your kindly reminding. We have answered this question in preceding text of this text. We have added the new description at Page 11 line 301 to 302 in our revised manuscript.

-L. 325: "base" -> "based"

**AU:** Thank you for your guidance, we are sorry for our carelessness and revised this mistake. New descriptions at Page 12 line 331 in our revised manuscript.

-L. 328: "major of them" --> "most of them" or "a majority of them"

**AU:** Thank you for your suggestion. We have revised the statement in our manuscript. New descriptions at Page 12 line 333 in our revised manuscript.

-L. 379: remove "totally"

**AU:** Thank you for your suggestion. We have revised the statement in our manuscript. New descriptions at Page 14 line 387 in our revised manuscript.

-L. 416: "Further explore" --> "Further exploration"

**AU:** Thank you for your suggestion. We have revised the statement in our manuscript. New descriptions at Page 15 line 425 in our revised manuscript.

-L. 430: "It was..." --> "It is..."

**AU:** Thank you for your guidance, we are sorry for our carelessness and revised this mistake. New descriptions at Page 16 line 431 in our revised manuscript.

-L. 439: "clearly" --> "clear"

**AU:** Thank you for your guidance, we are sorry for our carelessness and revised this mistake. New descriptions at Page 16 line 439 in our revised manuscript.

-Using "reasonable" sound negative regarding the paper's results. I would not use this adjective to describe the hypothesis (especially twice), instead: "conservative", "cautious", "parsimonious",...

**AU:** Thank you for your guidance, we have changed "reasonable" to "conservative" in our manuscript. New descriptions at Page 17 line 460, 464 and 470 in our revised manuscript.

**Referee: 2**

**Comments to the Author**

**Comment:** The authors have addressed most of my comments.  I think the paper has substantially improved since the last stage.  There are a few additional minor comments with respect to authors new additions to the manuscript:

**AU:** Thank you very much for your positive comments. We follow your instructions and add new statements in the revised manuscript. We believe that we have improved our manuscript to respond your major concerns in current revised manuscript.

**Comment:** 1 - Carry over comment from last round: “What does R1-R5 imply? Where do they come from?”.  The authors clarified it for me, but this is still not clear in the manuscript.  The discussion jumps straight to R3.  I am also not clear where in Figure 1c is R1-R5 defined?

**AU:** Thank you for your pointing our mistake. To make the definition of R1-R5 clear to the readers, we have corrected the description in parts of methods and we have added the description in legends of Figure 2a. New descriptions at Page 8 line 205 to 209 and Page 21 line 624 to 627 in our revised manuscript.

**Comment:** 2 - Thank you for the addition of the new section “Classification of SVs based on two specific patterns”.  Can the authors expand “A” and “SA” the first time they use it in this section?

**AU:** Thank you for your guidance. We have added the expand description of “A” and “SA” when the first time they use it. New descriptions at Page 6 to 7 line 169-176 in our revised manuscript.

**Comment:** 3 - This is completely discretionary – as I was reading the paper, I would intuitively think A-A as Asian-Asian comparison and not Asian-Absent.  A different convention for present and absent (e.g., Ap for Asian present and Aa for Asian absent) will help readers follow the paper better.

**AU:** Thank you for your suggestion. As your suggestion, we have converted “A-A” and “A-P” to “A-a” and “A-p” in our revised manuscript.

**Comment:** 4 - Is there a reason the authors decided to remove the flowchart from Figure 1?  Flowcharts provide a good summary of the methods and with some language changes, the flowchart would be a good first part of the figure.  I recommend that authors keep that figure.

**AU:** Thank you for your suggestion. The detailed process has been provided in the materials and methods of last revised manuscript, and there were enough graphs in Figure 1, so we decided to remove the flowchart from Figure 1. As your suggestion, we have decided to revise and add this flowchart to Supplementary figure 1 in our revised manuscript. New descriptions at Page 5 line 128 in our revised manuscript.

**Comment:** 5 - All figures are generally missing axis labels.  Axis labels help readers quickly understand what the figure is describing.  E.g., Figure 1d – what do the x- and y-axes represent?

**AU:** Thank you for your remarkable guidance, we are sorry for our carelessness and add the axis labels in figures. we have added the axis labels for all Figures.

**Comment:** 6 - Figure 1 c – please describe in the figure legend (and preferably also in the figure) what “yes” and “no” at the bottom of the figure indicate.

**AU:** Thank you for your pointing our mistake. As your suggestion, we have added the description for in “yes” and “no” figure legend of Figure 1c. New descriptions at Page 21 line 616 to 618 in our revised manuscript.

**Comment:** 7 - In several places, the authors use “blasted” to describe BLAST searches.  It reads awkwardly (I don’t think it’s a word yet…) and I recommend rephrasing it.  E.g., line 357-358: “Further blasted pattern-related TEs to these subfamilies…” could be rephrased to something like “We locally aligned the pattern-related TEs to the subfamilies using BLAST…”.  For this line, please also specify the parameters that were used to verify that the resulting hits were significant.

**AU:** Thank you for your professional suggestion, we are sorry for our false description with “blasted”. We have changed new descriptions at Page 8 line 218, Page 12 line 336, and Page 13 line 364 to 369 in our revised manuscript. In addition, we have added the version and parameters of BLAST.

**Comment:** 8 - Figure 3’s legend requires rewording “The coordinates indicate the homologies between SVs and transposable elements…”.  Specifically, I recommend changing “homologies” to “confidence” or “similarity” as the coordinates (identity and coverage in this case) do not indicate homology.  We can infer the homology between the SV and the best hit based on these parameters and P-value, but on their own, sequence identity doesn’t state homology.

**AU:** Thank you for your professional guidance, we are sorry for our false description of Figure 3a’s figure legend. As your suggestion, we have reworded it and new descriptions at Page 7 line 171 in our revised manuscript.
